# The multifaceted functions of lactate and its translational potentials in cancer

**DOI:** 10.3389/fphar.2026.1881938

**Published:** 2026-09-02

**Authors:** Ping Wang, Xia Yang, Yunfei Yan, Qiang Fu, Jinxia Hu, Sen Xu, Xin Wang, Fei Jiao

**Affiliations:** 1 Department of Medical Oncology, Yantaishan Hospital Affiliated to Shandong Medical and Pharmaceutical University, Yantai, China; 2 Department of Medical Cardiology, Yantai Affiliated Hospital of Shandong Medical and Pharmaceutical University, Yantai, China; 3 Department of Biochemistry and Molecular Biology, Shandong Medical and Pharmaceutical University, Yantai, China; 4 School of Pharmacology, Institute of Aging Medicine, Shandong Medical and Pharmaceutical University, Yantai, China; 5 Department of Clinical Laboratory & Health Service Training, Shandong Medical and Pharmaceutical University Affiliated 970 Hospital of the PLA Joint Logistic Support Force, Yantai, China

**Keywords:** cancer, epigenetic, immunity, lactate, lactylation, metabolism, tumor microenvironment

## Abstract

As considered as a mere waste product historically, the biological functions of lactate have been overlooked for a long time. Over the past decades, accumulated data has implicated the multiple and substantial roles of lactate in physiological and pathological contexts, particularly in cancer. This review aims to provide a comprehensive overview about lactate biology in both health homeostasis and oncology. We first presented a systematic elaboration about lactate metabolism, including its property, production, transport, shuttling as well as sensing. Additionally, the multifaceted functions of lactate in tumor microenvironment were summarized to explore the underlying mechanisms during metabolic reprogramming, signaling molecule, immune modulation and epigenetic regulation. Furthermore, we assessed the current lactate-targeted strategies for cancer intervention, exploring the feasibility as therapeutic targets in clinical application. Finally, the unsolved challenges were outlined to propose illuminating directions for future investigations and accelerate its translation into clinical practice in this field.

## Introduction

For a long time, lactate has been traditionally considered as a mere metabolic waste mainly derived of glycolysis (Baltazar et al., 2020; Yu X. et al., 2024). However, a plethora of recent studies suggest its pivotal roles in regulating various physiopathological processes, such as cancer (Ippolito et al., 2019; Liu H. et al., 2024; Nguyen et al., 2025). It is the case that lactate accumulation in tumor microenvironment (TME) contributes to the extracellular acidification which is a metabolic hallmark of cancer. This phenomenon is much more familiar to the Warburg effect (also known as aerobic glycolysis or glycolytic reprogramming), which means tumor cells prefer to producing lactate for rapid energy generation even under adequate oxygen conditions (Fendt, 2024; Li Z. et al., 2024). Moreover, because of the multifaceted roles of lactate in epithelial-mesenchymal transition, migration, angiogenesis as well as immune evasion, many studies have shown that the amount of lactate is closely linked to the spread of cancer to distant sites (Chen S. et al., 2024). For instance, lactate concentrations were shown to be substantially greater in metastatic areas than in nonmetastatic sites in cervical and colorectal malignancies (Gao et al., 2022). Currently, much is known that lactate is associated with energy homeostasis, immunomodulation, signal transduction and epigenetic regulation, all of which can influence tumor growth, invasion, metastasis, angiogenesis, immune evasion and drug resistance (Wang et al., 2020; Jiang et al., 2024).

With the chemical formula CH_3_CH(OH)COOH, lactate is also referred to 2-hydroxypropionic acid because of the hydroxyl group (-OH) in its molecular structure. As an organic acid, lactate commonly exists in three stereoisomeric forms: D-lactate, L-lactate, and racemic mixture DL-lactate (Zhao et al., 2025). In mammals, L-lactate is the predominant form as the plasma ratio of D-lactate to L-lactate is estimated at approximately under physiological conditions (Vavricka et al., 2024). Here, we will focus on the L-lactate and designate as lactate. Given its pKa of 3.83, lactate exists in solution either as the undissociated form (i.e., lactic acid) under low pH conditions or as an ionic salt (i.e., sodium lactate) under higher pH conditions. Therefore, the majority of lactate has a negative charge under physiological environments.

The understanding of cellular lactate metabolism and its underlying functions has been recognized gradually in the past centuries. Initially, lactate has been considered as a harmful metabolic waste under hypoxic conditions, such as intensive exercise (van Hall, 2010; Emhoff et al., 2013). Later, it has been firmly demonstrated that lactate can serve as a direct or alternate source of energy, through incorporation into the tricarboxylic acid (TCA) cycle or conversion into glucose (gluconeogenesis) in the liver by the Cori cycle, respectively (Rabinowitz and Enerback, 2020). Furthermore, it has been observed that lactate can be transported across cells, tissues, and organs with autocrine, paracrine, and endocrine-like manners. This fact introduced the lactate shuttling theory, which not only strengthen the multifaceted roles of lactate in intracellular and extracellular signal transduction mediated by multiple proteins and receptors, but also tightly related to the metabolic reprogramming in diverse cells (Zhang Y. et al., 2024). More recently, beyond its metabolic role, lactate-induced lactylation revealed a novel mechanism of post-translation modification (PTM), a critical link between metabolic status and epigenetic regulation (Jing et al., 2025; Wan et al., 2025).

These major milestones in understanding lactate’s biological functions extremely extend our knowledge about metabolic homeostasis, reprogramming, symbiosis and so on. In the context of cancer, mounting body of evidence also showed that abnormal lactate-related metabolisms exacerbate the trajectory of tumor progression, which involved lactate production, shuttling, sensing and lactylation (Garcia-Canaveras et al., 2019; Apostolova and Pearce, 2022; Zhang X. et al., 2024). Therefore, the present review aims to make a comprehensive summary about the pleiotropic action of lactate in cancers. We first described its essential roles as energy source and involved processes in normal and cancer metabolism. Its effects on the components of TME, particularly about the function of immune cells, were further discussed. In addition, we also presented the progress of protein lactylation through epigenetic mechanisms. Finally, the advancement and underlying challenges about the novel approach in cancer treatment based on potential targets in lactate metabolism were explored. The review will provide new insight of lactate in tumor progression and highlight its valuable perspectives on cancer management.

### History of lactic acid/lactate biochemistry

**FIGURE 1:**
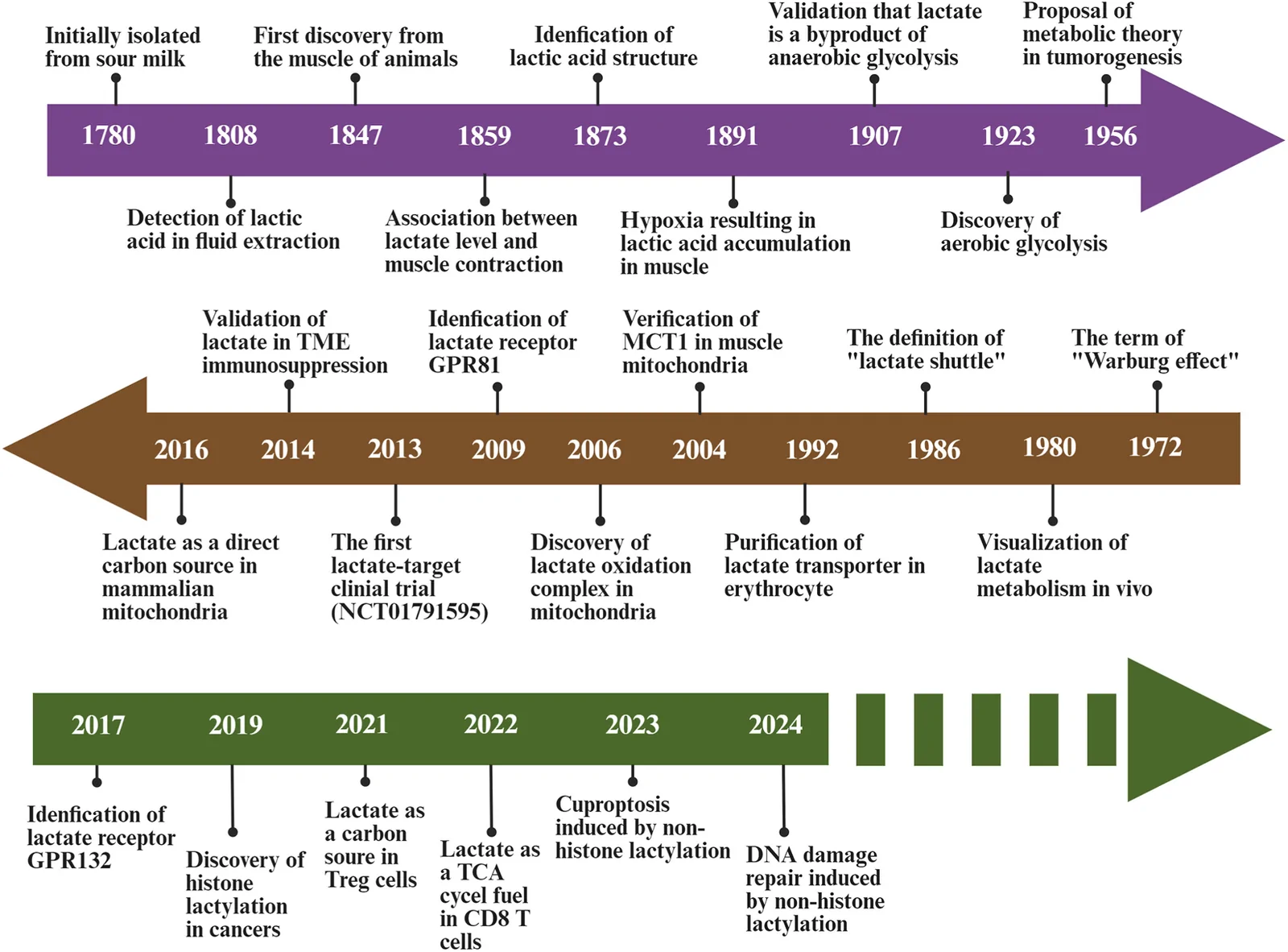
Timeline of some representative discoveries and breakthroughs in research of lactic acid. Figure created with BioRender (biorender.com). Abbreviations: CD, cluster of differentiation; GPR, G protein-coupled receptor; MCT, monocarboxylate transporter protein; Treg cell, regulatory T cell.

The discovery history of lactate is well documented and marks many groundbreaking milestones in biochemical and cancer research (Figure 1). In 1780, lactic acid was initially isolated from sour milk. Afterwards, lactate was detected in fluid extraction of meat and then further identified in the muscle of animals (Chen J. et al., 2024). In 1859, it has been confirmed that lactate level can significantly increase following intense exercise, indicating that lactate is tightly associated with energy metabolism in muscle contraction (Ma et al., 2025). Soon after, lactate in human blood under pathological conditions was further identified. With its structure was established in 1873, accumulating evidence showed that lactate is not only a metabolic buffer bridging oxidative phosphorylation (OXPHOS) and glycolysis, but also a metabolic fuel for most tissue cells, including malignant cells (Chen et al., 2025). Further studies unraveled that glutaminolysis can also contribute to lactate generation in cancers (Wang Z. H. et al., 2021). At present, it is well known that the Warburg effect in tumor cells is a central feature of cancer cell metabolism. This phenomenon was reported by Otto Warburg in 1927 and describes the preference of cancer cells to favor glycolysis for energy production, even under adequate oxygen conditions, leading to increased lactate synthesis. The term of “Warburg effect” has been formally put forward by Efraim Racker in 1972 (de la Cruz-Lopez et al., 2019). In the process of lactate study, another crucial finding is known as “lactate shuttle theory”, which was introduced in 1986. The “cell-cell lactate shuttle” and “intracellular lactate shuttle” concepts describe how lactate contributes to the transport of substrates for oxidation and gluconeogenesis, as well as in cellular signaling pathways, both within and between cells and tissues. More recently, a seminal PTM of lysine residues called lactylation (Kla) was discovered in 2019. Protein lactylation proposed as a new epigenetic modification not only opens up a novel dimension for the research of PTM regulation but also indicates a new insight for exploring lactate in pathophysiological processes, including metabolism, immunity, cancer and so on.

### Technical advancement of lactate/lactylation detection

Clinically, as the increase of lactate is usually related to tissue hypoxia and damage, lactate detection is an important tool in the evaluation of patient situations. Currently, arterial blood gas analysis serves as the gold standard for evaluating a patient’s oxygenation and ventilation conditions. If the detection does not require high accuracy, venous blood gas testing can be an alternative way. Both methods can be applied through point-of-care testing (POCT) devices, which can provide rapid clinical information and facilitate to suitable adjustments of treatment schemes for therapeutic benefit by fine-tune lactate metabolism (Collins, 2022). In the field of scientific and preclinical research, some new techniques, such as probe-based metabolic imaging, isotope-tracing systems as well as nuclear magnetic resonance spectroscopy (MRS), are implemented extensively to explore the biological properties and functions of lactate. For instance, a fluorescently tagged analogue of L-lactate was employed as a chemical probe for detecting lactate, aiming to investigate its transport and metabolic process within living cells (Rabuka et al., 2006). Moreover, through^13^C-labeled metabolic flux analysis, researchers are able to track the trajectory of each isotopically labeled carbon atom, which helps them acquire a more profound understanding of specific intermediates and the intricate metabolic processes caused by lactate (Befroy et al., 2014). Additionally, a high-performance imaging technique named FiLa was performed to achieve, real-time, quantitative dynamic, and *in situ* tracking of lactate metabolism in live cells, subcellular organelles, and *in vivo* (Wang A. et al., 2024). Novel advances from hyperpolarized (HP) ^13^C-MRI are changing the prospect and feasibility of measuring intracellular lactate concentration using as a tumor biomarker (Li et al., 2022). Positron emission tomography (PET) is another promising approach for metabolic imaging. It can reflect tumor glucose metabolism by imaging the radioactive glucose analog fluorine-18 fluorodeoxyglucose, a radiotracer which can alter metabolic activity based on glycolytic metabolism of cancer cells and enable diagnose and monitor tumors *in vivo* (Garg et al., 2025).

In the context of lactylation analysis, the major methods to detect lactylation include immunoblotting, mass spectrometry (MS) and computational prediction (Lu et al., 2024). Conceivably, immunoblot analysis utilizes antibodies that specifically recognize lactylation motifs to detect lactylation modifications in particular proteins through immunological reactions. The formation of cyclic ammonium ions of lactyl-lysine during high-performance liquid chromatography (HPLC)-tandem MS analysis serves as a new method for the identification of lysine lactylation (Wan et al., 2022). The progress in MS/MS-based proteomics techniques has facilitated the identification of numerous lactylated proteins and their modification sites across various species, which are also referred as lactylome (Yang et al., 2023). In addition, a chemical lactylation probe named YnLac has also been invented or characterizing protein lactylation in mammalian cells. Through the integration of YnLac, lactylated proteins can be directly labeled with fluorescent or alternative affinity markers to facilitate fluorescence visualization or proteomic analysis (Sun et al., 2022). In addition to conventional methods, computational prediction of Kla from protein sequences is an alternative approach to efficiently prioritize highly potential candidates for further experimental verification. For example, two novel computational models, Auto-Kla and DeepKla, were developed to predict protein lysine lactylation sites (Lv et al., 2022; Lai and Gao, 2023). With the growing availability of data and the iterative development of algorithm models, artificial intelligence (AI)-based analysis is increasingly being applied to predict and analyze lactylation (Jiang et al., 2021). Collectively, it can been anticipated that, with technology advances, more sensitive and high-throughput methods for detecting lactylations will offer novel insights about the impact of lactate/lactylationon biological processes of oncology.

### Lactate production: glyolysis and glutaminolysis

**FIGURE 2:**
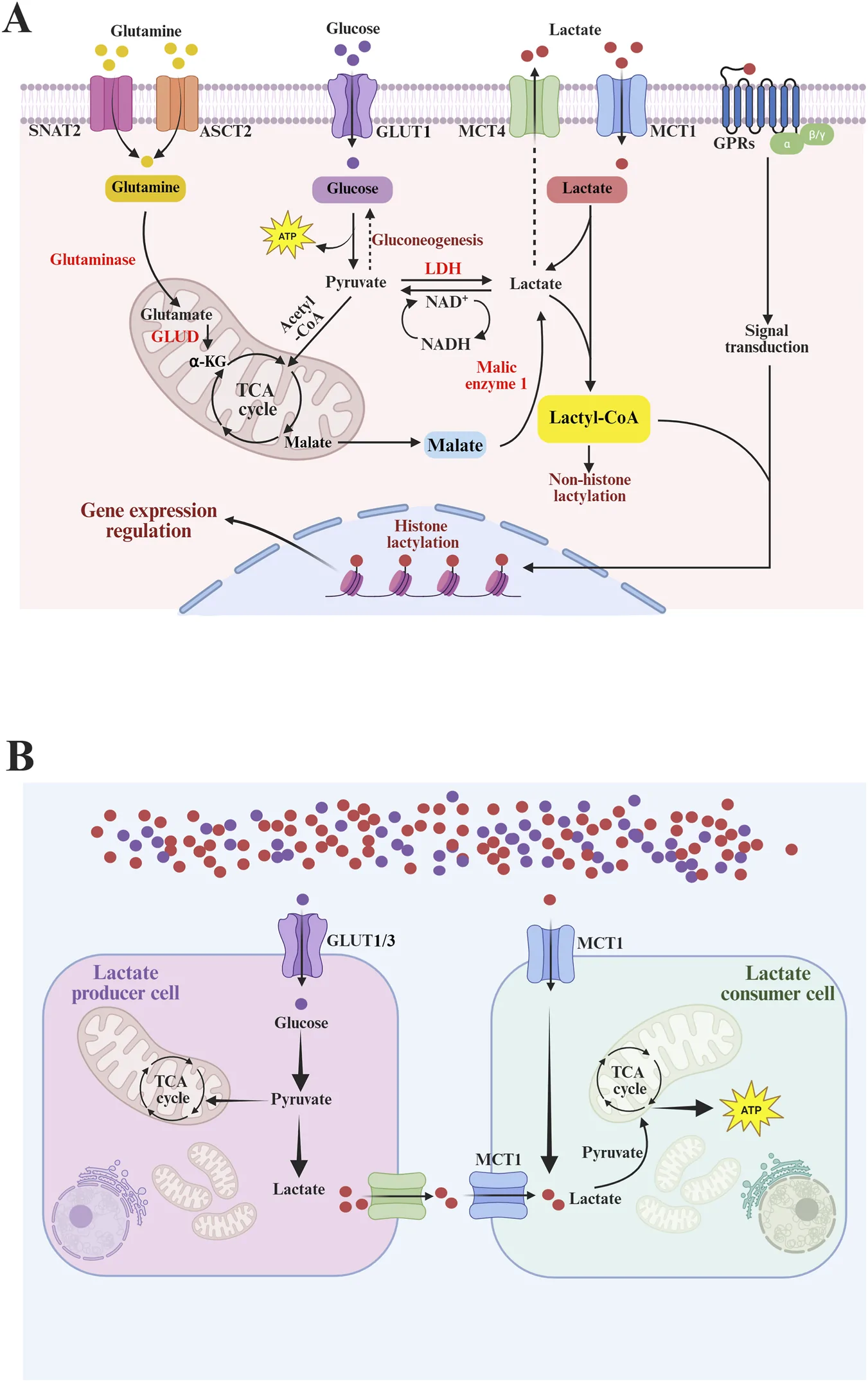
The illustration of lactate production, transport, signaling and shuttling. **(A)** The production, transport and signaling of lactate in cell. The main sources of intracellular lactate include glycolysis, glutaminolysis, and the uptake of exogenous lactate. To facilitate its rapid transport, influx and efflux of lactate can be regulated by MCT1 and MCT4, respectively. In addition to decomposition in mitochondria or gluconeogenesis in cytoplasm, a portion of lactate can act as a precursor of lactyl-CoA, which is involved in lactylation of histones and non-histone proteins. Extracellular lactate can also play signal transduction roles via GPR81 to regulate gene expression. **(B)** The shuttling of lactate between cells. In lactate producer cells, lactate can be produced through glycolysis in the cytoplasm and then excreted to extracellular space by MCT4.In lactate consumer cells, lactate can be uptaked and transported into mitochondria by MCT1, then oxidized to generate ATP. Figure created with BioRender (biorender.com). Abbreviations: ASCT2, type 2 amino acid transporter; ATP, adenosine 5′-triphosphate; GLUT, glucose transporter; GPR, G protein-coupled receptor; LDH, lactate dehydrogenase; MCT, monocarboxylate transporter; SNAT2, sodium-coupled neutral amino acid transporter 2; TCA, tricarboxylic acid.

In the mammalian cell, glycolysis and glutaminolysis are the two major pathways for lactate production (Figure 2A) (DeBerardinis et al., 2007). Glycolysis is the first stage of aerobic oxidation of glucose, in which pyruvic acid can be formed as an intermediate metabolite. When oxygen supply is sufficient, the generated pyruvic acid will enter the TCA to generate ATP via OXPHOS in mitochondria. On the contrary, pyruvate is reduced to lactate in the cytoplasm under hypoxic environment. This process is catalyzed by lactate dehydrogenase (LDH) with the oxidation of NADH to NAD^+^ simultaneously. LDH complex is a tetrameric enzyme composed of M and H subunits that can combine and form five homo or hetero tetramers in human tissues: from LDH1 to LDH5, respectively. LDH5 (also known as LDHA) preferentially reduces pyruvate to lactate, causing an increase in lactate production. In contrast, LDH1 (also known as LDHB) has a greater affinity for lactate than pyruvate, thus favoring the conversion of lactate to pyruvate (He et al., 2024). Regarding the interconnected metabolic reactions, pyruvate primarily obtained from glucose (65%) during the interconversion of pyruvate and lactate (Manosalva et al., 2021). It has been reported that the physiological ratio of lactate/pyruvate is approximately 10:1 due to a well-defined catalysis rate of LDH, implying that lactate is an important energy source for many tissues (e.g., erythrocytes, muscle and brain) to cope with physiological processes. Although glycolysis produces much less ATP compared to OXPHOS, the rate of glucose metabolism into lactate is 10-100times faster than the complete oxidation of glucose in the mitochondria. Therefore, the amount of ATP produced by glycolysis and OXPHOS is comparable within a given period of time (Looyens et al., 2021). Finally, it should be mentioned that LDH isoforms can provide additional diagnostic and/or prognostic information in pathological conditions, because of their characteristics of tissue-specific distribution. In context of cancer, although the assay of LDH isoforms is not yet a routine clinical practice to date, there are indeed a few studies to investigate the role of distinct LDH isoforms in various cancers (Claps et al., 2022).

As glutamine makes up over 20% of the free amino acid pool in plasma, its degradation (also referred to as glutaminolysis) is another source of lactate, especially in tumor cells (Romero-Garcia et al., 2016). During this pathway, a series of reactions are involved by the catalysis of diverse enzymes to make the conversion of glutamine to lactate. Briefly, glutamine can first be transported from the mitochondria to the cytosol under the regulation of c-Myc via several specific transporters, such as the type 2 amino acid transporter (ASCT2) and the sodium-coupled neutral amino acid transporter (SNAT2). Then, glutaminase and glutamate dehydrogenase contribute to the synthesis of α-ketoglutaric acid, which can enter the TCA cycle until pyruvate is produced (Gong et al., 2022). To a lesser extent, pyruvate can also be formed through alanine, serine, threonine, and cysteine catabolism (Perriello et al., 1995). Although it is now widely acknowledged that elevated lactate level and the subsequent acidification of TME drive various key oncogenic processes, the specific roles of glucose and glutamine in contributing to extracellular lactate in tumor still require further elucidation. For example, it would be intriguing to determine whether lactate derived from glutamine plays a part in tumor metabolic symbiosis (Zheng et al., 2025).

Under physiological conditions, it is estimated that various organs in the body can produce about 1500 mmol of lactate or 20 mmol/kg of body weight daily. With the homeostasis of lactate production and clearance, the normal lactate level in blood is approximately 1 mmol/L. Considering circulation has efficient buffering mechanisms to quickly remove lactate from the production site, lactate in moderate amounts is not harmful to the body as it can be efficiently eliminated by the liver (60%), kidneys (30%), and other organs (Looyens et al., 2021). However, its excessive accumulation in serum is catastrophic because of lactic acidosis, which has closely association with many physiological and pathological processes (Liu et al., 2025).

### Lactate transport, shuttling and sensing

Because of its hydrophilicity, lactate itself can hardly cross the plasma membrane by free diffusion. Hence, its role in cellular metabolism is highly dependent on specific transport mechanism, a process mediated by monocarboxylate transporters (MCTs) (Ruan et al., 2025). MCTs belong to solute carrier (SLC) transporter superfamily, which consists of more than 400 members of protein membrane transporters. Among them, MCT1-4 (also known as SLC16a1, SLC16a7, SLC16a8, and SLC16a3, respectively) are involved in lactate transport and expressed in different tissues. As the primary transporters responsible for lactate uptake and export, MCT1 is expressed in all cells induced by c-Myc and is responsible for the influx of lactate from the extracellular space, whereas MCT4 is highly expressed in glycolytic tissues and cancer cells induced by hypoxia and facilitates the efflux of lactate from the intracellular space (Certo et al., 2022; Li et al., 2025). In addition, two sodium-coupled lactate cotransporters named SMCT1 and SMCT2 (also known as SLC5A8 and SLC5A12, respectively) have also been identified to mediate transmembrane transfer of lactate. Although SMCTs differ from MCTs in terms of structure and mechanism, they still share some substrates with MCTs, including lactate (Manoharan et al., 2021).

Since 1985, the concept of lactate shuttle was introduced and has been continuously developed and refined in the past decades. Currently, it is proposed that the lactate shuttle transports lactate as a vehicle connecting glycolytic and oxidative metabolism within and between cells (Fiaschi et al., 2012; Sun et al., 2020). In this context, lactate shuttling can take two forms: intracellular and intercellular. Intracellular shuttling primarily occurs between the cell membrane and mitochondria, which is driven by a concentration or pH gradient or by redox state and prevailingly facilitated by mitochondrial LDH (mLDH) and mitochondrial lactate transporters. In contrast, intercellular shuttling is generated in glycolytic cells (producer) and then released into interstitial fluid or circulation to reach oxidative cells (consumer), where it is metabolized. Lactate shuttling occurs in many physiological and pathological conditions. Similarly, the shuttling of lactate between cell populations in TME is a vital phenomenon of tumor biology and is crucial for tumor initiation, progression, and metastasis (Brooks, 2018). This mechanism of lactate shuttling in cancer cells is also defined as metabolic symbiosis (Nakajima and Van Houten, 2013). Conceivably, lactate shuttling highlights the multifaceted functions of lactate in energy metabolism and signal transduction, providing novel therapeutic perspectives on lactate’s roles in disease.

Beyond an intermediate metabolite, lactic acid is also a type of signal transduction molecule, which can be sensed in various biological processes. Its signaling functions are mediated by its bidirectional shuttle between cells or intracellular organelles aforementioned, leading to a phenomenon called “lactormone” (Wu D. et al., 2023). In addition to the MCTs mentioned above, this signaling function depends on lactate receptors as well. It has been observed that lactate can serve as an endogenous ligand to interact with a class of G protein-coupled receptors (GPRs) on the cell membrane to modulate cellular behavior. As these GPRs are sensitive to the changes of pH, and can be activated under specific pH values, they act as a bridge between lactate signaling and microenvironmental pH variations (Fang et al., 2024). Among these GPRs, GPR81, also known as hydroxycarboxylic acid receptor 1 (HCAR-1), is the most intensively studied lactate receptor and is the primary receptor responsible for sensing and transmitting lactate signals (Li Y. et al., 2024). GPR132, which is also referred to as G2 accumulation protein (G2A), plays a role in sensing lactate and promoting signal transduction in cancers. Studies showed that GPR132 is a stress-inducible receptor whose functions include cell cycle, proliferation and immunity (Hernandez-Olmos et al., 2024). Furthermore, the other important mechanism of lactate-signaling pathway to influence cell functions is via lactylation, a novel type of PTM which will be highlighted further in the following section (Wang et al., 2023; Wu H. et al., 2023). The common transport, shuttling and sensing of lactate were illustrated in Figure 2B.

### Lactate-induced lactylation

In 2019, lactylation was first discovered on lysine residues of human H3 and H4 histones by the team of Zhang et al. (2019). Since then, growing number of histone Kla modification sites have been discovered. Further studies showed that lactylation is not limited solely to histones. Rather, it is widely distributed across a range of non-histone proteins as well (Yu H. et al., 2024). At present, the term “lactylation” is now characterized by the incorporation of lactate into lysine residues on both histones and non-histone proteins, in which the lactyl group is thought to attach to proteins via lactyl-coenzyme A (lactyl-CoA) or a nonenzymatic mechanism (Fu et al., 2022). The representative lactylation sites identified of histone and nonhistone in human were shown in Table 1. In recent years, lactylation has been detected in many proteins distributed in multiple cellular compartments, including cytoplasm, nucleus, mitochondria, endoplasmic reticulum, and cell membrane, which underscores the extensive impact of lactylation on cellular processes (Yang et al., 2022). In the context of cancer, the novel discovery of lactylation has also attracted considerable attention and has become one of the most prominent topics (Qu et al., 2023). For instance, increasing evidence demonstrated the pivotal role of lactylation in diverse cancers, including lung adenocarcinoma, gastric cancer, colorectal cancer, bladder cancer, breast cancer and so on (Ma et al., 2026). Moreover, crucial roles of lactylation in tumor progression have been highlighted, such as tumor initiation, abnormal proliferation, local invasion, distant metastasis and resistance (Fang et al., 2026).

**TABLE 1:** The representative lactylation sites of histone and non-histone identified in human.

Type	Protein	Kla site	Function
Histone	H3	K18	-Cell differentiation and metabolic reprogramming -Promote tumor progression -Potential biomarkers for diagnosing -Regulating effect of CD8 T cell functions -Aggravates microvascular anomalies -Promotes resistance to bevacizumab/cisplatin treatment
	H3	K18,K23	-Involves embryonic development
	H3	K9	-Regulates CD8 T cell effector functions -Promotes muscle generated -Promotes temozolomide resistance in glioma
	H3	K9,K14,K56	-Promotes liver cancer cell proliferation and metabolic reprogramming
	H4	K12	-Biomarker for Triple-negative breast cancer -Promotes anaplastic thyroid carcinoma -Promotes inflammation -Accelerates DNA replication and the cell cycle
	H4	K9	-Loading efficiency -Structural stability -Insufficient yield
	H4	K5,K8	-Regulation of telomerase activity
	H4	K5,K8,K12	-Promote tumor progression and drug resistance
Non-histone	HIF1α	Unknown	-Promotes angiogenesis and vasculogenic mimicry
	p53	K120, K129	-Contributes to tumorigenesis
	PKM2	K62	-Regulates macrophage phenotype transition
	AK2	K28	-Facilitates tumor cell proliferation and metastasis
	MRE11	K673	-Enhances homologous recombination repair
	METTL3	K281, K345	-Strengthens immunosuppression of myeloid cells
	MOESIN	K72	- Enhances TGF-β signaling in Treg cells
	YY1	K183	-Upregulates FGF2 transcription -Promotes angiogenesis
	eEF1A2	K408	-Promotes tumorigenesis
	ACSF2	K182	-Results in mitochondrial dysfunction
	KZF1	K164	-Promotes TH17 differentiation
	FASN	K673	-Mediates hepatic lipid accumulation
	NMNAT1	K128	-Promotes cancer growth

H histone; K: lysine.

Available evidence showed that lactylation modifications are intricate and multifaceted, involving complex processes. For enzymatic lactylation, specific enzymes and proteins are involved in the regulation of its activation, maintenance, and removal, in which lactylases (writers), delactylases (erasers), and lactylation recognition enzymes (readers) are capable of the functions of adding lactyl groups to histones or non-histones, eliminating lactyl groups from them, and identifying lactylation modifications, respectively (Chen A. N. et al., 2021). Given the structural resemblance between lactyl and acetyl groups, the enzymes first identified as writers and erasers for lactylation turned out to be the same ones that also function in acetylation processes. For instance, the P300 and its homolog CREB-binding protein (CBP) are such lactylation writer proteins. Presently, CBP and p300 were often studied together and referred to as the CBP/p300 complex because of their sequence homology and functional overlap (Gong et al., 2024). Further research has identified several other lactylation writers, including histone acetyl transferase (HAT), males absent on the first (MOF), lysine acetyl transferase 8 (KAT8), α-tubulin acetyltransferase 1 (ATAT1), and general control non-depressible 5 (GCN5) (Cheng and Guo, 2025). These writers utilize lactyl-CoA as a direct substrate to transfers the lactyl group to lysine residues on target proteins, leading to lactylation modification. The other manner of enzymatic lactylation involves aminoacyl-tRNA synthetase 1 (AARS1) or AARS2. Both enzymes are capable of directly transforming lactate and ATP into lactate-AMP, followed by the transfer of the lactyl moiety to lysine residues on target proteins (Zhu et al., 2025). For nonenzymatic lactylation, in contrast, lactyl-glutathione could act as a donor for acyl groups in protein lactylation independent of specific enzymes (Gaffney et al., 2020). Though it has been reported that the nonenzymatic mechanism highly depends on environmental factors like pH, temperature, and substrate concentration, the current research on this topic is relatively limited. Generally, H3K18 is the most extensively studied site for Kla. With the advancements in lactylation, other histone lactylation sites with regulatory functions have been reported, including H3K9, H3K14, H3K23, H3K56, H4K5, H4K80, and H4K12 (Wang W. et al., 2024). These histone modifications can alter chromatin structure to regulate gene transcription and expression. Moreover, lactate can also induce lactylation to influence protein stability, activity, interactions, and localization in non-histone proteins, such as transcription factors and metabolic enzymes (Xu et al., 2024). Therefore, lactylation has significant importance in modulating gene expression and altering the structure and functionality of proteins, consequently impacting vital cellular processes associated with pathophysiological processes. The common lactylation modification sites, functions and mechanism in cancers were shown in Table 2.

**TABLE 2:** The common lactylation modification sites, functions and mechanism in cancers.

Tumor types	Protein	Kla site	Mechanisms and effects
Esophageal cancer	Histone	H3K9	-LAMC2 transcription↑ -The proliferation and invasion of ESCC↑
	Non-histone	HIF1α	- KIAA1199 transcription↑ - angiogenesis↑
	Non-histone	Axin	-Ubiquitination of Axin1↑ -Anti-glycolytic function↑
	Non-histone	SHMT2	-MTHFD1L expression↑ -EC malignant progression↑
Gastric cancer	Histone	H3K18	- VCAM1 transcription↑ -Activates AKT-mTOR signaling pathway -Tumor cell proliferation, EMT and metastasis↑
	Non-histone	METTL16 K229	-Copper death↑
	Non-histone	CBX3 K10	-Tumor proliferation and growth↑
NSCLC	Histone	H3K18	- Enhances immune evasion via the POM121/MYC/PD-L1 pathway
	Histone	H4K12	- Pemetrexed resistance↑
	Non-histone	SOX9	-Stemness, migration, and invasion↑
	Non-histone	APOC2 K70	-Tumor metastasis↑ - Immunotherapy resistance↑
	Non-histone	IGF1R	-Tumor proliferation↑ -Metabolic reprogramming↑
Breast cancer	Histone	H3K18	-c-Myc↑ -Cancer progression↑
	Histone	H4K12	-A novel biomarker for TNBC
	Non-histone	p53 K120,K139	-Tumorigenesis↑
HCC	Histone	H3K18	-Transcription of IRS1↑ -HCC cell proliferation, migration and invasion↑
	Histone	H3K9,H3K56	-Malignant phenotype, proliferation, and metastasis↑
	Non-histone	c-Myc	-Stability of the c-myc protein↓
	Non-histone	GPC3	-Cell growth, stemness, and glycolysis↑
	Non-histone	MOESIN K72	-Immunosuppressive function↑ -HCC progression↑
	Non-histone	PKM2 K505	-Immunosuppressive function↑ -HCC metastasis↑
	Non-histone	AK2 K28	-Cell proliferation, invasion and metastasis↑
	Non-histone	CCNE2	-Proliferation, migration, and invasion↑
	Non-histone	CENPA K124	-HCC progression↑
Colorectal cancer	Histone	H3K18	-H3K18la-GPR37-Hippo pathway-liver metastases -METTL3-JaK1m6A-STAT3-Immunosuppression -Exacerbates resistance to bevacizumab treatment -CRC progression and metastasis↑ -Macrophage protumor function↑
​	Histone	H4K8	-LINC00152 expression↑ -CRC invasion and migration↑
	Non-histone	β-catenin	-Promotes proliferation through the Wnt pathway
	Non-histone	RIG-I	-Immunosuppressive activity of Tregs↑ -Antitumor activity of CD8^+^ T cells↓
	Non-histone	eEF1A2 K408	-Promotes CRC development
Cervical cancer	Histone	H3K18	-Promotes the reprogramming of TAMs
	Non-histone	PPP1R14B K140	-Proliferation and migration↑
	Non-histone	G6PD K45	-Cell proliferation↑
Prostate cancer	Histone	H3K18	-Neuroendocrine prostate cancer progression↑
	Non-histone	p53	-Prostate cancer progression↑
	Non-histone	CNPY3 K215 and K224	-Prostate cancer progression↑
Glioma	Histone	H3K18	-Self-renewal in glioma cells↑ -M2 macrophage polarization↑ -Progression of gliomas↑
	Histone	H3K9	-Resistance to temozolomide in the gliomas↑
	Non-histone	PTBP1 K436	-Glycolysis↑ -Exacerbation of tumorigenesis↑
	Non-histone	XRCC1 K247	-Treatment resistance↑
	Non-histone	VEGFR2 and VE-cadherin	-Angiogenic mimicry↑
Bladder cancer	Histone	H3K18	-Hippo pathway↑ -Cisplatin resistance↑

H histone; K: lysine. IRS1: insulin receptor substrate 1; TAMs: tumor-associated macrophages.

**FIGURE 3:**
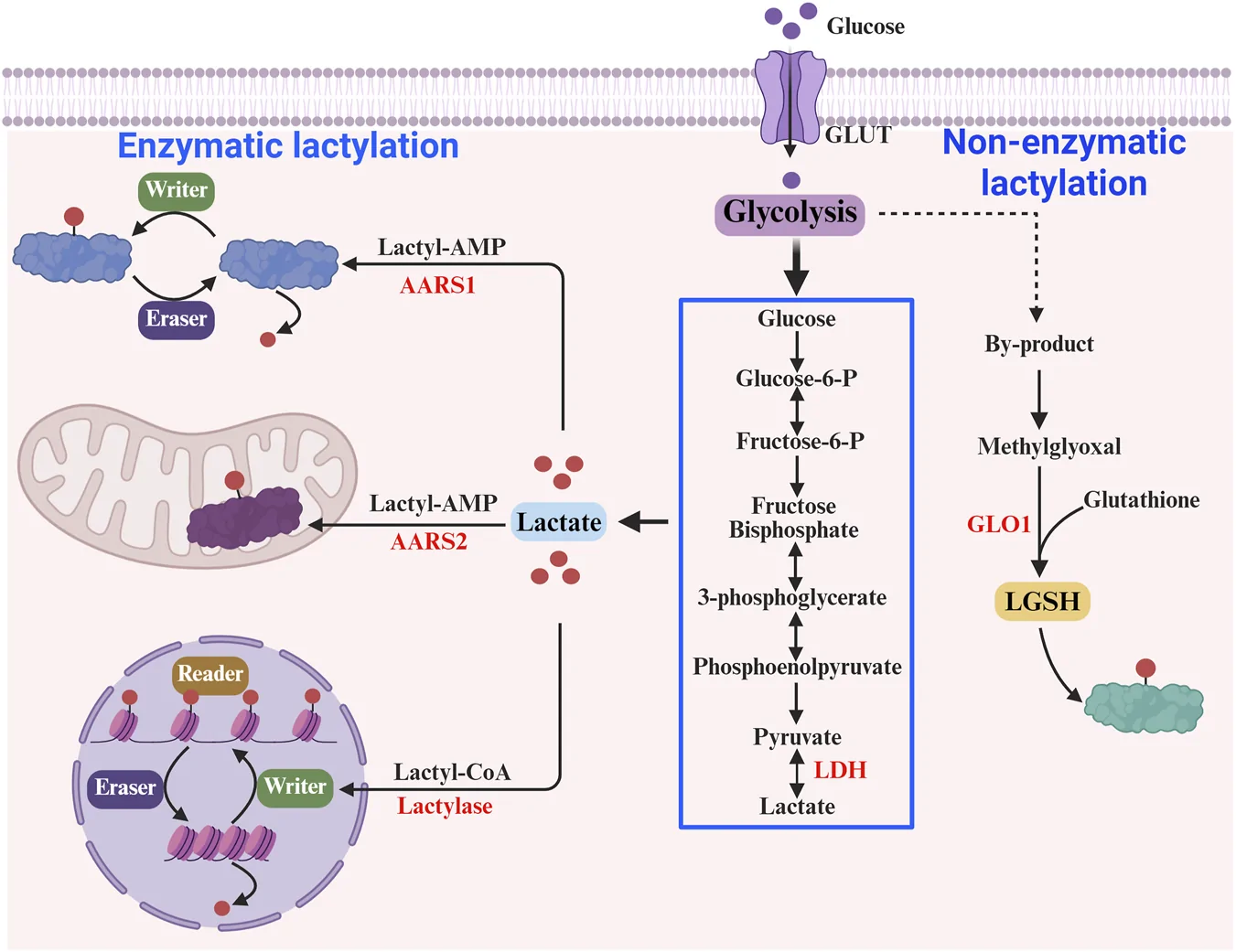
Enzymatic and nonenzymatic processes of lactylation modification. Depending on the different precursors (e.g., lactyl-CoA, lactyl-AMP and lactoyl-glutathione), the lactylation process can be divided into enzymatic or nonenzymatic reactions. Enzymatic reactions can take place in the nucleus, cytoplasm and mitochondria. During this process, different enzymes (e.g., writer, reader and eraser) are involved in the lactylation of histones and non-histone proteins. In contrast, non-enzymatic reactions can occur in the cytoplasm. During this process, methylglyoxal can be converted into lactoyl-glutathione, which is involved in the lactylation of target proteins. Figure created with BioRender (biorender.com). Abbreviations: AARS, aminoacyl-tRNA synthetase; GLO1, glyoxalase 1; GLUT, glucose transporter; LDH, lactate dehydrogenase; LGSH, lactoyl-glutathione.

Readers are defined as effector proteins or domains that have the ability to recognize and bind to lactylation groups. Although it had been speculated that readers associated with lactylation might encompass lactate-specific receptors, domains specific to lactylation, and other elements, the number of confirmed readers for lactylation remains quite limited. To date, Brg1 (or Smarca4) is the first recognized lactylation reader, which can bind to H3K18la (Hao et al., 2024). In addition, the readers, such as Dux and TRIM33, have also been reported to recognize Kla changes (Hu X. et al., 2024; Nunez et al., 2024). Because reader can play vital roles in signal transduction, protein subcellular localization, and protein-protein interactions by interacting with lactylation groups, more studies are needed to identify potential readers of lactylation. Lactylation is a reversible process, and research demonstrated that erasers for delactylation involved histone deacetylases (HDACs) and Sirtuins (SIRT) (Moreno-Yruela et al., 2022; Fan et al., 2023). It is the subtle collaboration between writers and erasers to enable cells can respond to changing environmental and metabolic conditions. The illustration of enzymatic and nonenzymatic lactylation was shown in Figure 3.

The discovery of lactylation opens up a new field for understanding how lactate changes cell functions, while many important scientific questions still need to be addressed. The most important issue is the potential synergistic or competitive relation about acetylation and lactylation. First, the substrates for these two epigenetic modifications are converted from pyruvate, a common precursor, through different metabolic pathways. Lactylation relies primarily on lactic acid from glycolysis, while acetylation primarily uses acetyl-CoA as a substrate from TCA cycle. Any factors affecting these metabolic activities may lead to the imbalance between lactylation and acetylation (Stacpoole and Dirain, 2024). It can be anticipated that some feedback and equilibrium mechanisms must exist between these two PTMs. Second, since both lactylation and acetylation are concurrently regulated by HATs and HDACs, it is necessary to ascertain the possible interaction. It is reported that the ability of HATs and HDACs to distinguish between acetyl-CoA and lactyl-CoA mainly hinges on factors such as enzyme structure, the specificity of the active site, the affinity of the chemical environment, and differences in substrate kinetics. For instance, research has indicated that the cellular concentration of lactyl-CoA is approximately 1/1000 of that of acetyl-CoA. Moreover, the time required for acetylation to achieve a steady state (6 h) is notably less than that for lactylation (24 h). This evidence suggests that lactylation has unique time dynamics compared to those of acetylation, even if they share some catalytic enzymes (Zhang et al., 2019; Varner et al., 2020). Third, both acetylation and lactylation predominantly take place at lysine residues. When proteins undergo these modifications, the sites of modification frequently overlap, further suggesting potential competitive or antagonistic effects between the two modifications. This crosstalk between acetylation and lactylation allows them to interact with each other to make a fine tuning of the structure, function and activity of proteins. Finally, as a connection of metabolism and epigenetic regulation, lactate-induce lactylation showed a time-dependent pattern, known as lactate clock. This interesting phenomenon has been observed in diverse physiological process, such as immunity, cell proliferation and blood vessel formation, which are highly associated with tumorigenesis and cancer progression (Zhang et al., 2019). However, proper level of lactylation during the lactate clock needs to be determined further. Together, the relationship between these two PTMs is worth exploring in depth, as lactylation and acetylation not only potentially influence each other in complex regulatory networks within the cell, but also may play synergistic or antagonistic roles in a wide range of biological processes.

### Metabolic reprogramming in cancer-beyond Warburg effect

The Warburg effect can result in enhanced lactate secretion and elevated acidification in the extracellular environment of tumor, a hallmark of cancer metabolic reprogramming. Both lactate and acidification profoundly influence the phenotype and functional attributes of cancer cells. Studies showed that, compared to OXPHOS, the rate of ATP production through glycolysis is approximately 30–200 times faster (Shestov et al., 2014; Vaupel et al., 2019). Correspondingly, one of the most significant roles of Warburg effect is to meet the high energy demands of cancer cells through the more rapid ATP generation. At the meanwhile, glycolytic reprogramming promotes the formation of diverse intermediates during cancer metabolism, which can serve as essential building blocks of lipids, nucleotides, and other biomolecules required for tumor proliferation (Barba et al., 2024). Moreover, lactate can act as a signaling molecule through multiple receptors and pathways to promote tumor initiation and progression. For instance, the NF-κβ pathway or the PI3K-AKT-CREB pathway can be activated by the lactate in tumor cells (Qian et al., 2021). Therefore, lactate is regarded as a crucial “oncometabolite” that exerts significant influences on cancer biology, both directly and by causing acidification of the TME.

Metabolic reprogramming of tumor cell is a complex process involving multi-dimension and multi-factor interactions, including a wide range of genetic and epigenetic abnormality. In detail, mounting data showed that the overproduction of lactate in cancer cells was mainly attributed to an imbalance in three regulatory factors: HIF-1α (hypoxia-inducible factor 1α), C-MYC and P53. As the growth of tumor leads to limited oxygen diffusion, cancer cells will respond to these environmental changes by upregulating HIF-1α, an important transcription factor. Thereby, HIF activates genes encoding key enzymes related to the glycolytic pathway, such as hexokinase 2 (HK2), 6-phosphofructo-2-kinase/fructose-2,6-biphosphatase 3 (PFKFB3), pyruvate kinase muscle isoform 2 (PKM2) and LDH. The upregulation of these glycolytic proteins results in the increase of glucose uptake, acceleration of glucose breakdown and lactate production. Interestingly, studies have provided evidence indicating that elevated glycolytic activity is linked to the activation of the mechanistic target of rapamycin (mTOR) signaling pathway in cancer cells. In turn, the activation of the mTOR pathway can also increase the expression of HIF-1α, thereby further facilitating lactate production in cancer cells (Hua et al., 2019; Sun et al., 2025). Additionally, it has been proved that overproduction of lactate in cancer cells can be regulated by C-MYC via the induction of LDH5 activity, the inhibition of LDH1 activity, as well as the increase of glutamine uptake. Furthermore, the metabolism of cancer cells is also subject to the influence of tumor suppressor gene activity. Studies have shown that the absence of the p53 protein inhibits the expression and synthesis of the cytochrome c oxidase gene, leading to the reduction of mitochondrial respiration and facilitating the Warburg effect (Wanka et al., 2012). Last but not least, it should be highlighted that the lactate-induced lactylation has obvious influence on these regulatory proteins as well, which greatly increased the complex relationship between lactate production and tumor characteristics. For instance, Kla of HIF-1α can stabilize itself under normal hypoxic conditions and enhance the transcription of angiogenesis-related genes (Wang J. et al., 2024). Similarly, the function of p53 can be impaired by lactic acid produced by tumors, particularly via the lactylation of K120 and K139 residues (Zong et al., 2024).

The understanding of cancer related to the Warburg effect became more profound and comprehensive in recent years. It is well known that tumor heterogeneity and plasticity involved multiple factors and potential mechanisms, such as oxygen availability. As tumor growth is much faster than angiogenesis, cancer cells adjacent to microvessels are normoxic due to sufficient oxygen, while those remote to bloodstreams are hypoxic because of the absence of oxygen supply (Chen et al., 2022). In this context, a number of researchers proposed that normoxic tumor cells can take up lactate derived from hypoxic or glycolytic cells (e.g., tumor-associated fibroblasts, tumor-associated macrophages and glycolytic tumor cells) in TME. In this process, lactate can be reutilized by different cancer cell populations, a phenomenon called reverse Warburg effect (Marciniak and Wagner, 2023; Vavricka et al., 2024; Wang J. et al., 2024). This observation demonstrates that glycolysis is not an absolute reliance for all tumors, as certain tumors show a strong dependence on OXPHOS and consequently of mitochondrial function. Therefore, reverse Warburg effect can be regarded as a novel model of metabolic symbiosis to facilitate the survival of both normoxic and hypoxic tumor cells under acidic conditions.

### Immunomodulation of lactate in TME

**FIGURE 4:**
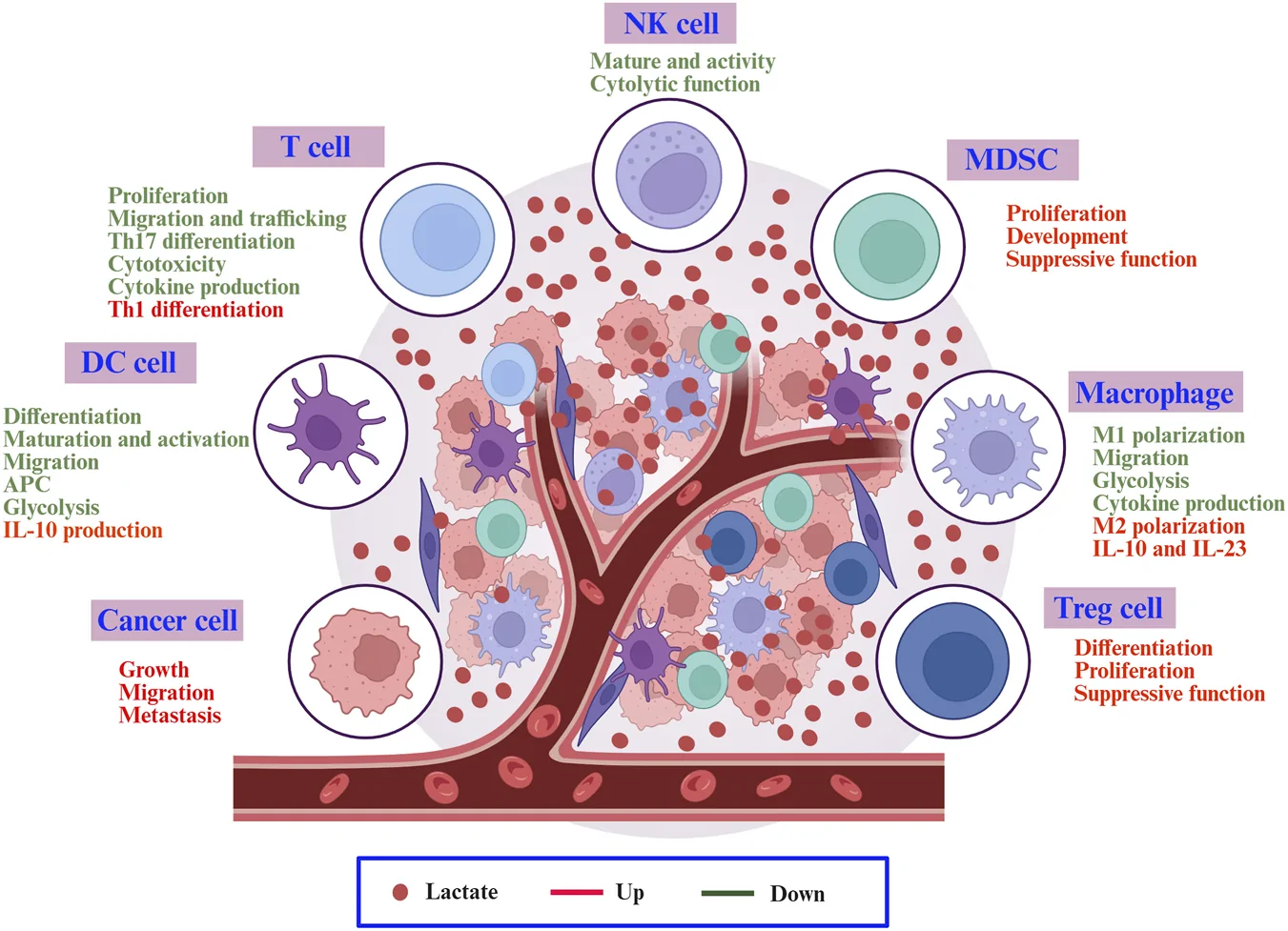
Immunomodulatory effects of lactate in the tumor microenvironment. The tumor microenvironment consists of a variety of cell populations, including tumor, stromal, immune cells, and etc. Lactate in the tumor microenvironment can modulate the innate and adaptive immune response in a cell-specific manner. It suppresses the differentiation, activation and migration of DCs, leading to the inhibition of cytotoxic T cells, reducing the proliferation, migration and trafficking of T cells. Lactate inhibits the maturation and activity of NK cells and facilitates the suppressive function of Treg cells. Additionally, lactate potentiates the M2 conversion and suppresses M1 polarization of macrophages, promoting angiogenesis and tumorigenesis. Conversely, it promotes the maintenance of immunosuppressive function in MDSCs in an acidic environment, contributing to tumor progression. Figure created with BioRender (biorender.com). Abbreviations: DC, dendritic cell; MDSC, myeloid-derived suppressor cell; NK, natural killer cell; Treg, regulatory T cell.

TME is a key component of the multidimensional cancer ecosystems, which is composed of biological microenvironment and abiotic factors. The TME is an intricate and dynamic network covering tumor cells, immune cells, stromal cells, extracellular matrix, blood vessels, as well as metabolites and signaling molecules (Binnewies et al., 2018; Baghban et al., 2020). It should be noted that the hematological TME exhibits distinct structural and functional features compared to that of solid tumors. In solid tumors, the TME is shaped by the surrounding tissue architecture, resulting in spatially restricted compartments. In contrast, the TME in hematological malignancies is more dynamic and spatially diffuse due to the shortage of rigid compartmentalization. This fluidity facilitates systemic dissemination, immune modulation, and therapeutic resistance. Here, we mainly focus on the TME of solid tumor (Walid et al., 2026). For the last decades, considerable research has focused on the mechanisms behind immune-metabolism crosstalk. In terms of lactate metabolism, its role of immunomodulation in the TME is much more intricate than we previously anticipated. Considering the dual effects of lactate on different immune cells in TME, the elucidation of potential mechanism is of great interest for its clinical translation for cancer treatment, such as improving the efficacy of cancer immunotherapy (Hao et al., 2024; Wang W. et al., 2024). The immunomodulation of lactate in TME was shown in Figure 4.

#### Tumor-associated macrophages

Tumor-associated macrophages (TAMs) are a major population of tumor infiltrating leukocytes abounding in the cancer stroma with various characteristics and functions. As TAMs are preferentially located in the hypoxic region of the tumor, they can promote angiogenesis and metastasis via the transcription of VEGF, bFGF, PDGF and prostaglandin E2 which are induced by high expression of HIF-1α. Also, they can generate proteases to degrade the extracellular matrix (ECM) in the TME, thereby, promote the migration and diffusion of cancer cells (Zhou et al., 2022). According to the tumor-promoting function of TAMs, researchers grouped macrophages into M1-subset and M2-subset. M1 macrophages enable antigen presentation and immune factor activation, exhibiting anti-tumor features. Conversely, M2 macrophages suppress inflammation, increase tumor immune surveillance evasion, and promote tumor growth and metastasis (Tao et al., 2023). Metabolically, the M1 phenotypes utilize aerobic glycolysis, whereas M2 phenotypes exhibit activation of OXPHOS and fatty acid oxidation (FAO) (Batista-Gonzalez et al., 2019). Under normal physiological circumstances, a state of dynamic equilibrium exists between M1 and M2 macrophages. In contrast, elevated lactate from glycolysis is essential for maintaining the protumoral activities of TAMs.

Emerging evidence has unveiled some mechanisms through which lactate influences macrophage function via both receptor-dependent and receptor-independent signaling pathways. GPR81 is the most intensively studied lactate receptor and its expression is depending on cell type and tissue microenvironment. For instance, it is reported that macrophages located in the intestines and lungs demonstrate elevated expression levels of GPR81 when compared to their counterparts in the spleen (Tao et al., 2023). GPR81 signaling in macrophages exerts inhibitory influences on the NF-κB and yes-associated protein (YAP) pathways by activating AMP-activated protein kinase (AMPK) and large tumor suppressor kinases (LATS). In the TME, GPR81 signaling, which is crucial for immune suppression against tumors, can influence the induction of regulatory antigen-presenting cells (APCs) and the upregulation of programmed death-ligand 1 (PD-L1). Furthermore, lactate-GPR81 signaling regulates the expression of MCT1 and MCT4 in tumor cells, playing a vital role in the energy provision for these cells (Feng et al., 2017). GPR132 is also expressed at high levels on the surface of macrophages (Zhang et al., 2022). The GPR132 receptor present on the macrophage surface triggers the activation of multiple downstream signaling pathways linked to immune regulation, including cAMP, protein kinase A (PKA), and ERK. In turn, these pathways subsequently promote the adhesion, migration, and invasion of cancer cells (Zeng et al., 2020). In addition to these receptor-dependent manners, extracellular lactate can also modulate the APC’s functions by directly regulating the activation of multiple signaling pathways and transcription factors after getting transported into the cells through MCTs and SMCTs (Weiss and Angiari, 2020). Moreover, Zhang and colleagues demonstrated that the H3K18la modification at the promoter during M1 polarization can directly promote the transcription of homeostatic genes (Zhang et al., 2019). Nonetheless, further study is warranted to elucidate the potential mechanism through which lactylation regulates the transcription of target genes. Together, lactate in TME induces the polarization of TAMs through its effects on transcription, signaling cascades, and epigenetic alterations, ultimately promoting tumor advancement.

#### Dendritic cells

As the most important APCs, dendritic cells (DCs) can efficiently intake, process, and present antigen information to T cells which are essential to the antitumor immune response. Undoubtedly, potent DC capacity is required for sufficient T-cell activation (Wculek et al., 2020). Studies revealed that lactate has pivotal and multifaceted effects on DC functions, involving differentiation, maturation, migration and antigen presentation. First, lactate-induced acidosis impairs monocyte’s differentiation into DCs. It is observed that exorbitant lactate levels in the TME (i.e., 40 mM) can make significant inhibition of DCs differentiation and maturation. Lactate can also impede the differentiation of DCs by triggering the production of IL-10, which concurrently leads to a reduction in IL-12 secretion and CD1a expression through the stimulation by Toll-like receptors (TLRs) (Gottfried et al., 2006). Secondly, the movement of DCs to secondary lymphoid organs and tissues plays a crucial role in initiating adaptive immune reactions and conducting tumor immune surveillance. The migration of DCs relies on the specific expression of chemokine receptors on their surface and the corresponding chemokine ligands present in the tissues and draining lymph nodes (DLNs). In these locations, DCs prime and activate T cells, thereby triggering adaptive immune responses (Palucka and Banchereau, 2012). *Ex vivo* studies have shown that high lactate levels (20 mM) from glycolytic metabolism can influence the oligomerization of CCR7, a chemokine receptor required for DC migration (Guak et al., 2018). Furthermore, lactate in the TME has the ability to modulate DC migration by influencing the expression levels of several pivotal glycolytic enzymes (Peng et al., 2021). Finally, emerging studies have found several mechanisms associated with the suppressive role of lactate in antigen presentation of DCs. For antigen presentation and cross-priming T cells, DCs require some membrane trafficking proteins to act as key regulators, such as SNARE (soluble n-ethylmaleimide sensitive factor attachment protein receptor) and VAMP3 (vesicle-associated membrane protein 3). Evidence showed that lactate can directly inhibit these proteins in DCs results in impaired cross-presentation of antigens associated with tumors (Caslin et al., 2021). In addition, the acidic TME speeds up the degradation of antigens within DCs, compromises their chemotactic capacity, and consequently impedes the immune system’s ability to conduct surveillance on tumors. Similarly, GPR81 located on the surface of plasmacytoid dendritic cells (pDCs) is capable of detecting lactate, leading to a reduction in antigen presentation (Raychaudhuri et al., 2019).

#### T cells

T cells are capable of detecting fluctuations in lactate levels and triggering intracellular signaling pathways, which in turn regulate cellular function and maintain homeostasis. As there are several excellent reviews discussed extensively how lactate shapes the functions of T cells, here we will only make a discussion briefly. In general, lactate has the capacity to influence T-cell-mediated immune reactions by functioning in a dual capacity, serving both as a metabolic substrate and as a signaling molecule. In quiescent state, CD8^+^ and CD4^+^ T cells only express low levels of MCT1 (Renner et al., 2019). When T cells are activated through the interaction between CD28 and the B7 receptor, there is a significant increase in MCT1 expression, and concurrently, the metabolic profile of T cells shifts from OXPHOS to glycolysis (Frauwirth et al., 2002). The metabolic shift of T cells in the TME brings out the outcome that activated T cells not only have to compete with tumor cells for glucose, but also cause an endogenous lactate accumulation, which hampers the antitumor activity of effector T cells. Additionally, lactic acidosis triggers T-cell apoptosis as well, as it decreases NAD^+^ levels, which leads to alterations in NAD^+^-dependent enzymatic processes and a consequent reduction in glycolytic intermediates necessary for cell proliferation (Quinn et al., 2020). At the same time, lactate serves as a crucial metabolic substrate and enables regulatory T cells (Treg) to flourish in the high-lactate, low-glucose conditions of the TME because of the metabolic flexibility of Treg cells (Angelin et al., 2017).

In addition to the direct metabolic effects, intracellular signal pathways will feedback to lactate level to regulate functions of diverse T cells. For instance, it has been observed that lactic acidosis can impair the functions of cytotoxic T lymphocytes (CTLs) by inhibiting T-cell receptor-triggered activation of the p38 and JNK/c-JUN pathways, which are important for IFN-α production (Mendler et al., 2012). Furthermore, lactate has the ability to modulate the polarization of CD4^+^ T cells and decrease the proportion of T helper 1 (Th1) cell subsets. It achieves this by triggering SIRT1-mediated deacetylation and degradation of T-bet transcription factors (Comito et al., 2019). Also, lactate-mediated signaling within CD4^+^ T cells promotes the differentiation towards Th17 cells while inhibiting the migration and trafficking of T cells (Pucino et al., 2019). In terms of Treg cells, lactate assumes a significant role in boosting their inhibitory functions by triggering pivotal signaling cascades, such as PI3K/Akt/mTOR pathway, and by elevating the expression levels of FOXP3. Additionally, the recruitment of Tregs can be promoted by lactate in the TME to facilitate Treg infiltration via upregulating chemokines such as CXCL12 and CX3CL1, which contributes to the immunosuppressive environment (Su et al., 2024). Finally, the pivotal role of lactylation should not be overlooked during the maintenance of immune homeostasis, in which several mechanisms are involved, including influence on Treg cells, the disruption of Th17/Treg balance, as well as the effectiveness of CTLs.

#### Other immune cells

Natural killer (NK) cells are innate immune cells that can secrete cytokines, perforin, and granzyme and display potent antitumor effects. Studies showed that high lactate concentrations affect NK cytotoxic activity by intracellular acidification and inducing apoptosis of NK cells. In melanoma mouse models, researchers found that lactate in TME decreased the release of lytic granule contents, such as perforin and granzymes (Brand et al., 2016). Furthermore, lactate exerts an inhibitory effect on the activation of nuclear factor of activated T cells (NFAT) in NK cells. As a result, it suppresses the generation of IFN-γ and TNF-α, ultimately leading to a reduction in the cytotoxic reaction against tumor cells. Conversely, it was observed that NK cells exhibited heightened activation and degranulation, along with increased expressions of perforin and CD107a, due to the buffering of lactic acid and the reversal of TME acidity (Long et al., 2018). In addition to these direct suppressions of NK cells, elevated lactate also indirectly restrains NK cells by increasing the number of myeloid-derived suppressor cells (MDSCs). MDSCs are the most prominent cell population in immunosuppressive immune cells. It has been found that increased glycolysis and elevated lactate concentrations can induce the development and expansion frequency of MDSCs (Li et al., 2018). Moreover, MDSCs can also been activated by lactate signaling through GPR81/mTOR/HIF-1α/STAT3 pathway, establishing a tumor immunosuppressive microenvironment to favor tumor growth (Yang et al., 2020).

### Lactacte -targeting in cancer treatments

**FIGURE 5:**
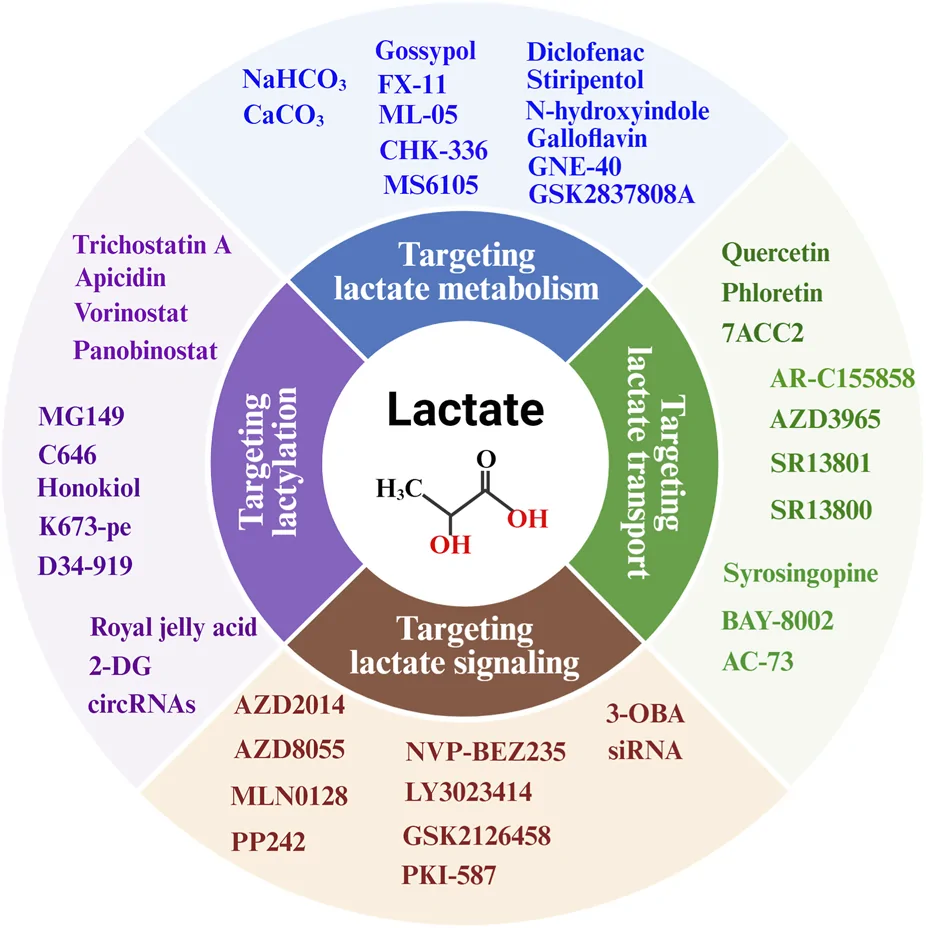
Pharmacological molecules targeting lactate/lactylation intervention in cancer and other diseases. Potential pharmacological molecules or drugs under exploration were illustrated. These therapeutic strategies are being investigated to target different pathways of lactate, such as metabolism, transport, signaling as well as lactylation. Figure created with BioRender (biorender.com).

Given that the disruption of lactate homeostasis poses a significant obstacle in the process of cancer progression, there exists substantial interest in targeting lactate metabolism and its signaling pathways for therapeutic purposes. Currently, promising approaches targeting lactate metabolism and signaling are performed to improve the efficacy of tumor therapy. All in all, available strategies include targeting lactate metabolism (e.g., LDH inhibitors), lactate transporters (e.g., MCT inhibitors) and signaling (e.g., GPR81/GPR132 antagonists), as well as synergizing with other conventional cancer treatments such as chemotherapy, radiotherapy and immunotherapy (Manoharan et al., 2021; Chen et al., 2025). In this section, we will make a discussion about the preclinical studies and clinical trials of these pharmacological molecules (Table 3; Figure 5).

**TABLE 3:** The therapeutic inhibitors targeting the lactate/lactylation in cancers.

Drug	Target	Mechanism	Applications	Status/Trial No.
Oxamate	LDHA	Lactate production	Prostate cancer, melanoma, and NSCLC	Pre-clinical
GSK2837808A	LDHA	Lactate production	Liver cancer	Pre-clinical
Stiripentol	LDHA	Lactate production	Peritoneal metastatic carcinoma	ChicTR2400083649
Gossypol (AT-101) and its derivative, e.g., FX-11	LDHA	Lactate production	Adrenocortical carcinoma, chronic lymphocytic leukemia, breast cancer, prostate cancer, and lymphoma	NCT00848016, NCT00286780, NCT06133088, NCT00286806, NCT05338931
CHK-336	LDHA	Lactate production	Healthy volunteers	NCT05367661
ML-05	LDHA	Lactate production	Pancreatic cancer, breast cancer, melanoma	Pre-clinical
GNE-40	LDHA	Lactate production	Pancreatic cancer	Pre-clinical
LOX	Lactate	Lactate decomposition	HCC, melanoma, breast cancer, GBM	Pre-clinical
NaHCO_3_, CaCO_3_	Lactate	Lactate decomposition	Brease cancer, NSCLC	Pre-clinical
AZD3965	MCT1	Lactate uptake	Solid tumor, lymphoma	NCT01791595
AR-C155858 (SR13801)	MCT1	Lactate uptake	Breast cancer, CRC	Pre-clinical
AR-C122982 (SR13800)	MCT1	Lactate uptake	Lymphoma, Neuroblastoma, breast cancer	Pre-clinical
BAY-8002	MCT1	Lactate uptake	Triple negative breast cancer	Pre-clinical
7ACC2	MCT1	Lactate uptake	Pancreatic cancer	Pre-clinical
Syrosingopine	MCT1/4	Lactate transport	HCC, lung cancer, breast cancer	Pre-clinical
AC-73	CD147	Lactate transport	HCC, AML	Pre-clinical
Temsirolimus, everolimus AZD2014, AZD8055, GSK2126458	mTOR	Lactate signaling	Bladder cancer, breast cancer, HCC	NCT00805129, NCT01597388, NCT00999882, NCT00972686
3-OBA	GPR81	Lactate signaling	CRC	NCT05338307
MG149	HDAC	Lactylation	CRC	Pre-clinical
C646	p300	Lactylation	Thyroid cancer	Pre-clinical
Honokiol	SIRT3	Lactylation	HCC	Pre-clinical
K673-pe	MRE11	Lactylation	CRC	Pre-clinical
D34-919	XRCC1	Lactylation	GBM	Pre-clinical
β-alanine	AARS1	Lactylation	CRC	Pre-clinical
Royal jelly acid	-	Glycolysis, lactylation	Liver tumor	Pre-clinical
CircXRN2 CircATXN7	Circular RNA	Lactylation	Bladder cancer	Pre-clinical
2-DG	Hexokinase	Glycolysis	Prostate cancer, lung cancer Breast cancer	NCT00096707 NCT00588185

Abbreviations: NSCLC: non-small cell lung cancer; HCC: hepatocellular carcinoma; AML: acute myeloid leukemia; CRC: colorectal cancer; GBM: glioblastoma; LDHA: lactate dehydrogenase A; MCT: monocarboxylate transporter; HDAC: histone deacetylase; 3-OBA: 3-hydroxybutyrate; 2-DG: 2-deoxy-D-glucose; LOX: lactate oxidase; mTOR: mechanistic target of rapamycin; SIRT, 3: Sirtuin 3; AARS1: aminoacyl-tRNA, synthetase 1; XRCC1: X-ray repair cross-complementing 1; MRE11: meiotic recombination 11.

#### Targeting lactate and its metabolism

Concerning the inhibitory role of lactate in tumor, it is plausible and practical for cancer treatment through the direct elimination lactate from TME. One of the logical strategies is based on the hypothesis about neutralizing lactate with basic salts, such as NaHCO_3_ and CaCO_3_. Indeed, the administration of NaHCO_3_ was applied in mouse models of metastatic breast cancer to inhibit spontaneous metastases (Robey et al., 2009). Similarly, CaCO_3_ nanoparticles were performed to suppress the proliferation and migration of breast cancer cells via maintaining the pH within the normal physiological range (Lam et al., 2021). Notably, another complementary approach known as alkalization therapy was advocated by some pioneering researchers to improve the effectiveness of conventional treatments. Briefly, alkalization therapy is defined as systemic alkalization of the body using alkalizing diet and agents to increase the pH of the local TME. This therapy strategy is based on the fact that current cancer treatments often leave the TME acidic, resulting in poor therapeutic efficacy and severe side effects. In general, although some efficacy in diverse cancers has observed, its clinical applications for anticancer treatment are still limited. Further extensive research and clinical trials are warranted to accurately assess its potential and efficacy as a cancer treatment strategy, involving the development of standard protocol for alkalization therapy, the establishment of accurate methods to measure the pH of the TME, as well as more reliable clinical evidence (Wada et al., 2022; Bahrami H and Greiner, 2023). Finally, it should be emphasized that pH regulation with alkalization must remain within physiologically appropriate limits, as either acidosis or excessive alkalinity can both result in pathological stress and disturb the normal cellular functions.

However, direct removal of lactate from TME is challenging and usually infeasible. Therefore, current strategies mainly focus on indirectly reducing lactate concentrations by inhibiting its generation. The popular method is through the inhibition of LDHA, which contributes to the primary lactate production in cancer (Gao et al., 2022). To date, various inhibitors of LDHA with different mechanisms were tested in preclinical studies and even in clinical trials. Oxamate, a pyruvate analog binding to LDHA, can prevent the conversion of pyruvate to lactate and has been reported to inhibit gastric cancer cell proliferation (Hashimoto et al., 2025). NADH-competitive LDHA inhibitors, including gossypol (also named AT-101) and FX11 (derivative of gossypol) have also been demonstrated to inhibit the proliferation of cancer cells and impede the progression of cancer (Sharma et al., 2025). Gossypol has advanced to phase I/II clinical trials aimed at treating adrenocortical carcinoma, chronic lymphocytic leukemia, advanced HR-positive and HER2-negative breast cancer, and metastatic castration-resistant prostate cancer (Xie et al., 2019). Positive outcomes have been observed in high-risk patients, as well as in certain patients experiencing extended periods of progression-free survival or overall survival (NCT00848016, NCT00286780andNCT06133088) (Renner et al., 2022). ML-05, a novel inhibitor targeting LDHA, significantly reduces lactate production, which in turn not only suppresses tumor growth but also boosts the antitumor immune activity of CD8^+^ T cells (Du et al., 2022). Furthermore, CHK-336, an orally administered LDHA inhibitor, has undergone evaluation regarding its tolerability, safety, and pharmacokinetic characteristics in healthy individuals (NCT05367661) (Zhang T. et al., 2024). Moreover, MS6105, a LDH Proteolysis Targeting Chimera (PROTAC) degrader, represents a new class of small-molecule inhibitors that target lactate/lactylation processes through the ubiquitin-proteasome pathway. PROTAC technology has effectively enabled the simultaneous targeting of both LDHA and LDHB, leading to the inhibition of proliferation across various pancreatic cancer cell lines (Sun et al., 2023). Other LDHA inhibitors have also been reported to inhibit tumor proliferation, such as diclofenac, stiripentol, N-hydroxyindole, galloflavin and GNE-40 (Sharma et al., 2022). Notably, LDH-targeted therapy also improves curative effect when combined with other methods of cancer treatments. For instance, in humanized mouse NSCLC model, the combination of oxamate and pembrolizumab (PD-1 monoclonal antibody) stimulated CD8^+^ T cell infiltration and hindered tumor proliferation, thereby sensitizing immunotherapy (Qiao et al., 2021). In preclinical melanoma mouse model, it has been confirmed that the efficacy of adoptive T cell therapy increased remarkably by the administration of GSK2837808A (LDHA inhibitor) (Cascone et al., 2018).

Another promising option to reduce lactate levels in TME involves lactate oxidation to pyruvate catalyzed by lactate oxidase (LOX) with the formation of hydrogen peroxide (H_2_O_2_) (Wu et al., 2021). In this context, LOX is encapsulated through cationic polyethyleneimine (PEI) and copper ions (Cu^2+^), in which lactate can be trapped by the cationic PEI component actively and then degraded by an encapsulated reservoir of LOX. During this process, Cu^2+^ ions function as a catalyst during the Fenton reaction, involved in the decomposition of H_2_O_2_. Interestingly, pyruvate generated from the reaction can in turn activate CRISPR/Cas9-mediated signal-regulatory protein alpha (SIRPα) genome-editing systems. Collaborated with a metal-organic framework, nanoparticle named LPZ can be further formed by LOX, Cas9/sgSIRPα plasmids and mannose-modified PEG loaded-ZIF-67. Studies showed that this method provides a way to directly edit macrophage genes at the tumor site and offers a promising tactic for boosting the effectiveness of immunotherapy (Li et al., 2023). More recently, LOX has been engineered to attach to the surface of *lactobacillus*, enhancing its ability to target lesions and improving delivery efficiency. This, in turn, activates the chemotoxic drug to trigger apoptosis through the complete oxidation of lactate and the depletion of intra-tumor oxygen catalyzed by LOX (Wu et al., 2022).

#### Targeting lactate transport

As aforementioned, MCT1/4 play central roles of in lactate transport and metabolic symbiosis associated with cancer development. Therefore, targeting MCT may be beneficial target for cancers resistant to conventional treatment. On the one hand, MCT1 inhibition can cause the apoptosis of hypoxic cancer cells that primarily rely on glucose for survival because of the competition of glucose between normoxic cancer cells and hypoxic cancer cells. On the other hand, MCT4 inhibition can result in intracellular acidosis and decreased survival of hypoxic cancer cells, because these cells cannot secrete excessive lactate over time (Wang Z. H. et al., 2021). At present, numerous inhibitors MCTs have demonstrated efficacy in preclinical trials. For instance, classical inhibitors of MCT1/MCT4 have been reported to exhibit effectiveness against various types of cancer, such as simvastatin, phloretin, lonidamine, quercetin, and alpha-cyano-4-hydroxycinnamate (CHC) (Wang Y. et al., 2021). However, these earliest MCT inhibitors with poor specificity largely limited their clinical application. Correspondingly, newer MCT inhibitors have higher selectivity for individual MCT isoforms than first-generation MCT inhibitors. For example, 7-(Nbenzyl-N-methylamino)-2-oxo-2H-chromene-3-carboxylic acid (7ACC2) is a potent inhibitor of MCT1 and mitochondrial pyruvate transport that has been reported to be effective in multiple human cancer types (Draoui et al., 2014). Other novel small-molecule inhibitors, including AZD3965, AR-C155858 (SR13801), AR-C122982 (SR13800), Syrosingopine and BAY-8002, have demonstrated potent MCT1-inhibitory activities. Among these therapeutic inhibitors, AZD3965 has completed phase I/II clinical trials for advanced prostate cancer, gastric cancer, or hematological tumors (NCT01791595), demonstrating promise as a cancer therapy (Halford et al., 2023). Last but not least, CD147 is also considered a promising target due to its vital function in modulating MCT expression. It achieves this by stabilizing MCTs and ensuring their localization to the cell membrane. Research has indicated that when CD147 function is disrupted by AC-73 (a specific inhibitor of CD147), the protein levels of MCT1 and MCT4 decline. This, in turn, leads to a reduction in lactate efflux and a suppression of tumor growth. However, it's important to highlight that CD147 is widely expressed and engages in interactions with various other proteins at the cell surface. Therefore, when devising strategies that target the interactions between CD147 and MCTs, it is essential to minimize drug toxicity (Spinello et al., 2019).

In addition to directly targeting cancer cells, the regulatory roles of MCT inhibitors in immune cells of the TME should not be overlooked. For example, since the uptake of lactate mediated by MCT1 is vital for Treg cells and TAMs to sustain their immunosuppressive and tumor-promoting capabilities, eliminating MCT1 in these cells hinders their buildup in the hypoxic and lactate-abundant TME. This creates a setting that is favorable for anti-tumor immunity. Hence, MCT1 inhibitors might exert their therapeutic actions by directly curbing tumor growth and simultaneously suppressing the functions of Treg cells and TAMs (Renner et al., 2019). Similarly, employing AR-C155858 to block lactate transport in pDCs restored the production of IFNα and enhanced the immune responses against 4T1 tumors in mice (Guan et al., 2019). Likewise, in severe combined immunodeficiency mice bearing Raji xenografts, AZD3965 resulted in the inhibition of tumor growth, accompanied by an increased infiltration of tumor immune cells, including DCs and NKs (Beloueche-Babari et al., 2020). Also, another study has demonstrated that suppression of MCT1 and MCT4 can restore T cell-induced immune function and boost the immune response to immune checkpoint inhibitors in melanoma patients (Renner et al., 2019).

#### Targeting lactate signaling

Due to its crucial function in tumorigenesis including its regulation of metabolism and stimulation of tumor progression, the mTOR pathway represents a highly potential target for cancer treatment. As its name termed, rapamycin acts as a specific inhibitor for mTOR pathway, resulting in decreased expression of HIF-1α and c-Myc, downregulation of glycolytic enzymes, thereby suppressing lactate production in cancer cells (Mossmann et al., 2018). Nevertheless, the limited bioavailability and solubility make rapamycin less effective in practice. Alternatively, various rapamycin analogues and inhibitors have been developed and utilized to treat multiple cancers, including ATP-competitive mTOR inhibitors (MLN0128, AZD2014, AZD8055 and PP242), rapamycin analogues (temsirolimus and everolimus), and dual PI3K/mTOR inhibitors (GSK2126458, LY3023414, NVP-BEZ235, and PKI-587) (Hua et al., 2019). Although the monotherapy of mTOR inhibitors had only limited antitumor effect, the combinational therapy with mTOR inhibition and other treatments (e.g., metformin or PD-1 antibody) showed synergistic antitumor activity (Li et al., 2017). Similarly, the combined effects of targeting the mTOR pathway and suppressing glycolysis have been documented in various types of cancers, such as hematologic malignancy and colorectal cancer (Xu et al., 2005; Xing et al., 2018).

Lactate receptors, such as GPR81/GPR132, exhibit increased expression in multiple cancer types and significantly contribute to the progression of tumors. In addition, preclinical studies demonstrated that these signalings in immune cells leads to the attenuation of antitumor immune responses. For example, Chen et al. reported that 3-OBA (3-hydroxybutyrate, a GPR81 inhibitor) and metformin can work together to limit cancer cell proliferation *in vitro* (Chen S. et al., 2021). Thus, the blockade of cognate GPR81/GPR132-mediated signalings represents emerging targets to suppress the proliferation of cancer cells while enhancing the antitumor immunity. Recently, in a 4T1 breast cancer model, Raychaudhuri and colleagues has shown that intratumoral injection of siRNA targeting GPR81 along with an MCT inhibitor resulted in a significant reduction in tumor growth (Raychaudhuri et al., 2019). Likewise, another study has confirmed that blocking GPR132 signaling in macrophages markedly reduced breast cancer progression and metastasis in mice. Pharmacological inhibition of GPR132 signaling had a similar effect on tumor burden and antitumor immune responses (Chen et al., 2017). In this context, further studies in understanding this lactate signaling networks will aid in the efficiency of cancer treatment.

#### Targeting lactylation

As a novel PTM, lactylation has garnered extensive attention in gene expression and oncology. Preliminary evidence revealed that lactylation could be a potential candidate for tumor therapy. Given that lactate is essential for lactylation, agents that target lactylation can generally be categorized into two types: those that directly interfere with the lactylation process and those that indirectly affect upstream metabolic pathways (He et al., 2024). First, the potential of HDAC inhibitors, which are also capable of influencing histone lactylation, have been investigated to affect cancer progression and immune evasion. For example, a great number of studies showed that prominent HDAC inhibitors, including trichostatin A, apicidin, vorinostat and panobinostat, have efficacy in various types of cancers by epigenetic regulations (Moreno-Yruela et al., 2022; He et al., 2025). Additionally, the suppression of the pan-Kla writer KAT8 through the application of the HDAC inhibitor MG149 disrupts the KAT8-eEF1A2 Kla pathway and inhibits the growth of colorectal cancer, especially within a lactic acid-rich TME (Xie et al., 2024). Nevertheless, the effects of HDAC inhibitors on lactylation are multifaceted, as these inhibitors may also enhance acetylation processes. In this context, modulation of certain writers or erasers more specific to lactylation seems to be more practical for cancer treatment. C646, an inhibitor of p300, showed enhanced anti-tumor effects when synergized with the targeted drug vemurafenib (a Raf inhibitor) (Cheng et al., 2019). Likewise, as a SIRT3 activator, honokiol has demonstrated anti-tumor effects by directly activating delactylation enzymes in hepatocellular carcinoma (Jin et al., 2023). In a study of liver cancer stem cells, it has been observed that inhibition of H3K9la and H3K56la by demethylzeylasteral decreased the tumorigenesis both *in vivo* and *in vitro* (Pan et al., 2022). In term of nonhistone proteins, K673-pe can inhibit the lactylation of MRE11 (meiotic recombination 11) K673 site and exhibit a synergistic tumor-suppressive effect when combined with chemotherapy in CRC (Chen Y. et al., 2024). Similarly, D34-919 can suppress the lactylation of XRCC1 (X-ray repair cross-complementing 1) through the blockade of aldehyde dehydrogenase 1 family member A3 (ALDH1A3) and PKM2, thereby restoring its sensitivity to chemotherapy and radiotherapy in glioblastoma (Sun et al., 2024). More recently, Zong and colleagues introduced a novel targeting approach focused on inhibiting AARS1, which acts as a bridge linking tumor cell metabolism to alterations in the proteome. In a mouse model of colorectal cancer, the administration of β-alanine disrupted the interaction between AARS1 and lactate, thereby halting the subsequent process of lactyl transfer. Consequently, the p53 protein was unable to undergo lactylation at positions K120 and K139, leading to the suppression of tumor progression (Zong et al., 2024).

Because lactylation process is highly relied on the lactate level, pharmacological molecules offer an indirect strategy to modulate lactylation via the inhibition of glycolysis or regulation of other metabolic pathways. For example, studies have revealed that Royal jelly acid, also known as 10-hydroxy-2-decenoic acid, can inhibit glycolysis-related metabolic pathways in liver cancer cells. This inhibition leads to a decrease in lactylation modifications at the H3K9la and H3K14la sites by suppressing the activity of LDH (Xu et al., 2023). Similarly, oxamate, which has been discussed above as a competitive inhibitor of LDH, has also been widely demonstrated to decrease lactylation levels. In addition to LDH, the suppression of hexokinase by 2-deoxyglucose (2-DG) is now being explored in combination therapy trials (NCT00096707) because of limited efficacy as a monotherapy (Lin et al., 2003). Beyond these traditional inhibitors, some oncogenic circular RNAs, such as CircXRN2 and CircATXN7, emerged as potential avenues targeting lactylation to achieve anti-tumor effects (Xie et al., 2023; Zhou et al., 2024). Collectively, a deeper understanding of lactylation mechanisms and targets in tumors is crucial for developing novel treatment strategies. Further research and clinical trials are required to translate these strategies into improvement of cancer treatment.

### Challenges and outlooks

The essential role of lactate and lactylation brings about increasing attention in tumor onset and progression, while new targets and opportunities can be anticipated for cancer treatment. However, the field of research remains in its early stages, leaving some critical challenges unsolved.

The first issue should be emphasized is that lactate can be produced by many normal tissues under physiological conditions, such as muscle cells undergoing intense anaerobic exercise. In other words, lactate is not exclusively a product of cancer cells. As a result, therapeutic strategies aimed at eliminating lactate-producing cells may face important biological dilemma which can potentially affect normal physiological processes and *vice versa*. For instance, on one hand, the increased levels of circulating lactate frequently reported in the context of cancer cachexia, a syndrome affecting 50%–80% of cancer patients and accounting for 20% of cancer-related death (Tan et al., 2026). Liu and his colleagues showed that lactate is not merely a pivotal mediator associated with the hypercatabolic phenotype in cancer, but also a characteristic metabolite in cancer cachexia (Liu X. et al., 2024). One the other hand, clinical and epidemiological studies consistently showed that regular physical activity or even high-intensity training is strongly correlated with a lower incidence of various cancers and obviously positive effects in cancer patients. The confusing paradox may be due to the different ways of lactate metabolism in exercise and cancer, including local or systemic location of lactate, the concentration and duration of lactate exposure, the presence or absence of a buffering environment, as well as its interactions with specific cell types. Further studies demonstrated that lactate derived from tumor seems to be pro-tumorigenic, driving immune suppression and disease progression, whereas short bursts of lactate from exercise can enhance anti-tumor immunity, highlighting the context-dependent effects that render lactate either beneficial or detrimental (Ahmadi Hekmatikar et al., 2025).

The second tough challenge is about the pleiotropic roles of lactate in the extremely complicated TME evolving various cell types and multiple signaling pathways. Though various therapeutic strategies targeting lactate metabolism and transport have been developed to exploit the key role of lactate in cancer metabolism and immune modulation, the clinical translation is still limited because of off-target effects and few well-designed clinical trials of lactate-targeting drugs (Hayes et al., 2021). For example, when intervening in lactate metabolism, it is crucial to guarantee that the method employed can efficiently eradicate cancer cells while preserving the normal functionality of healthy cells. Likewise, given that tumors from various patients may demonstrate significant heterogeneity and adaptability in their metabolic traits, a comprehensive understanding of how lactate influences treatment outcomes is necessary, along with the development of personalized strategies that account for the diverse metabolic profiles of tumors among different patients (Jiang et al., 2024).

Thirdly, studies available or ongoing most focused on L-lactate, while D-lactate has been largely overlooked. D-lactate is primarily produced by intestinal bacteria (Li et al., 2025). In specific metabolic and pathological scenarios, D-lactate exhibits a unique function when compared to L-lactate. Regarding the significant role of gut microbiota imbalance in some important pathological processes, it can be postulated that D-lactate metabolism and accumulation should be associated with many diseases (Fabian et al., 2017). For instance, the concentration of D-lactate rises notably in diverse types of cancer, especially gastrointestinal cancers, as a result of the modified gut microbiota that boosts its production. In addition, recent studies have shown that D-lactate can regulate M2 macrophages and reshape the immunosuppressive TME in hepatocellular carcinoma, suggesting its potential as a therapeutic target (Han et al., 2023). Furthermore, due to the its impact on mitochondrial dysfunction and oxidative stress, the involvement of D-lactate in metabolic diseases, such as type 2 diabetes and neurodegenerative disorders, has also been found (Chou et al., 2015). However, further research is required to uncover new insights into the physiological roles of D-lactate. In summary, as our understanding about L-lactate functions is extending and deepening, the exploration about the unique roles of D-lactate seems to be more imperative and important.

Finally, the great achievements in the realm of lactylation are exciting, while a considerable number of fundamental issues have not been elucidated. A report from Galligan et al. showed that the process of lactylation is directly influenced by the levels of lactyl-CoA, rather than by lactate concentration (Gaffney et al., 2020). This finding raises the question whether lactylation consistently accompanies elevated lactate levels despite of the correlation between lactate and lactylation. Currently, lactylation occurs at the site of lysine exclusively. In contrast, other potential sites beside lysine have been found in histone acetylation and succinylation (Wang G. et al., 2024). Therefore, whether other amino acids can be targeted for histone lactylation needs to ascertain. Similarly, for nonhistone lactylation, research at the molecular level should explore the specific effects of lactylation on protein structure, stability, and function, and how these effects are achieved through precise molecular mechanisms (Hu Y. et al., 2024). Moreover, recent studies have uncovered specific modifications involving L-lactate and D-lactate at particular protein sites. However, the functional dynamics-whether they act synergistically or antagonistically-between these two modifications, either at identical or distinct sites within the same protein, have yet to be investigated (Llibre et al., 2025). Moreover, lactylation in cells exhibits intricate interactions with other PTMs. The network of these PTMs is extremely complex in spatiotemporal dimensions, which is required to be investigated more intensively (Gou et al., 2025). Future research should shed light on the interactive competition and synergistic effects between these PTMs, as well as the precise mechanisms about the regulation of gene expression in different cell types and biological contexts. Such studies will not only help us understand the scientific basis of biology, but also provide potential targets for the development of novel therapeutic strategies, especially in the field of cancer. To resolve these issues, interdisciplinary collaboration is required to unravel the underlying mechanisms of lactylation in cellular function and disease management.

### Conclusion

In summary, lactate is becoming a novel research hotspot as an ancient metabolite. Far beyond only a byproduct of glycolysis, lactate forms tight links among cell metabolism, immune modulation and epigenetic regulation. These multifaceted roles of lactate position it as a pivotal player in both health homeostasis and cancer biology. Despite of great achievements in lactate biology, further elucidation of precise molecular mechanisms is needed and some significant challenges remain. It can be anticipated that future research with multidisciplinary collaboration will not only bring new insight into the functions of lactate in physiologic and pathological processes, but also provide a promising opportunity for its translation in cancer treatment.

## Glossary

2-DG: 2-deoxyglucose
7ACC2: 7-(Nbenzyl-N-methylamino)-2-oxo-2H-chromene-3-carboxylic acid
AARS: aminoacyl-tRNA synthetase
AI: artificial intelligence
ALDH1A3: aldehyde dehydrogenase 1 family member A3
AMPK: AMP-activated protein kinase
APC: antigen-presenting cell
ASCT2: type 2 amino acid transporter
ATAT1: α-tubulin acetyltransferase 1
CBP: CREB-binding protein
CHC: a-cyano-4-hydroxycinnamate
CTL: cytotoxic T lymphocyte
DC: dendritic cell
DLN: draining lymph node
ECM: extracellular matrix
FAO: fatty acid oxidation
HAT: histone acetyl transferase
HCAR-1: hydroxycarboxylic acid receptor 1
HDAC: histone deacetylase
HPLC: high-pressure liquid chromatography
GCN5: general control non-depressible 5
GPR: G protein-coupled receptor
HIF-1α: hypoxia-inducible factor 1α
HK2: hexokinase 2
KAT8: lysine acetyl transferase 8
Kla: lysine actylation
lactyl-CoA: lactyl-coenzyme A
LATS: large tumor suppressor kinase
LDH: lactate dehydrogenase
LOX: lactate oxidase
MCT: monocarboxylate transporter
MDSC: myeloid-derived suppressor cell
MOF: males absent on the first
MRE11: meiotic recombination 11
MRS: magnetic resonance spectroscopy
MS: mass spectrometry
mTOR: mechanistic target of rapamycin
NK: natural killer cell
NFAT: nuclear factor-activated T cell
OXPHOS: oxidative phosphorylation
pDC: plasmacytoid dendritic cell
PD-L1: programmed death-ligand 1
PEI: polyethyleneimine
PET: positron emission tomography
PFKFB3: 6-phosphofructo-2-kinase/fructose-2,6-biphosphatase 3
PKA: protein kinase A
PKM2: pyruvate kinase muscle isoform 2
POCT: point-of-care testing
PROTAC: proteolysis targeting chimeras
PTM: post-translation modification
SIRPα: signal-regulatory protein alpha
SIRT: sirtuin
SMCT: sodium-coupled lactate cotransporter
SNARE: soluble n-ethylmaleimide sensitive factor attachment protein receptor
SNAT2: sodium-coupled neutral amino acid transporter 2
TAM: tumor-associated macrophage
TCA: tricarboxylic acid
Th: T helper cell
TME: tumor microenvironment
TLR: Toll-like receptor
Treg: regulatory T cell
VAMP3: vesicle-associated membrane protein 3
XRCC1: X-ray repair cross-complementing 1
YAP: yes-associated protein

## References

[B1] Ahmadi Hekmatikar A. H. Zolfaghari G. Basereh A. Awang Daud D. M. Khoramipour K. (2025). Context matters: divergent roles of exercise-induced and tumor-derived lactate in cancer. Biomolecules 15 (7), 1010. 10.3390/biom15071010 40723880 PMC12293860

[B2] Angelin A. Gil-de-Gomez L. Dahiya S. Jiao J. Guo L. Levine M. H. (2017). Foxp3 reprograms T cell metabolism to function in low-glucose, high-lactate environments. Cell Metab. 25 (6), 1282–1293 e1287. 10.1016/j.cmet.2016.12.018 28416194 PMC5462872

[B3] Apostolova P. Pearce E. L. (2022). Lactic acid and lactate: revisiting the physiological roles in the tumor microenvironment. Trends Immunol. 43 (12), 969–977. 10.1016/j.it.2022.10.005 36319537 PMC10905416

[B4] Baghban R. Roshangar L. Jahanban-Esfahlan R. Seidi K. Ebrahimi-Kalan A. Jaymand M. (2020). Tumor microenvironment complexity and therapeutic implications at a glance. Cell Commun. Signal 18 (1), 59. 10.1186/s12964-020-0530-4 32264958 PMC7140346

[B5] Bahrami H T. M. Greiner T. (2023). A small-scale test of whether alkalizing diets can reduce the risk of disease as predicted by the warburg hypothesis. World Nutr. 14 (4), 3–15. 10.26596/wn.20231443-15

[B6] Baltazar F. Afonso J. Costa M. Granja S. (2020). Lactate beyond a waste metabolite: metabolic affairs and signaling in malignancy. Front. Oncol. 10, 231. 10.3389/fonc.2020.00231 32257942 PMC7093491

[B7] Barba I. Carrillo-Bosch L. Seoane J. (2024). Targeting the warburg effect in cancer: where do we stand? Int. J. Mol. Sci. 25 (6), 3142. 10.3390/ijms25063142 38542116 PMC10970388

[B8] Batista-Gonzalez A. Vidal R. Criollo A. Carreno L. J. (2019). New insights on the role of lipid metabolism in the metabolic reprogramming of macrophages. Front. Immunol. 10, 2993. 10.3389/fimmu.2019.02993 31998297 PMC6966486

[B9] Befroy D. E. Perry R. J. Jain N. Dufour S. Cline G. W. Trimmer J. K. (2014). Direct assessment of hepatic mitochondrial oxidative and anaplerotic fluxes in humans using dynamic 13C magnetic resonance spectroscopy. Nat. Med. 20 (1), 98–102. 10.1038/nm.3415 24317120 PMC3947269

[B10] Beloueche-Babari M. Casals Galobart T. Delgado-Goni T. Wantuch S. Parkes H. G. Tandy D. (2020). Monocarboxylate transporter 1 blockade with AZD3965 inhibits lipid biosynthesis and increases tumour immune cell infiltration. Br. J. Cancer 122 (6), 895–903. 10.1038/s41416-019-0717-x 31937921 PMC7078321

[B11] Binnewies M. Roberts E. W. Kersten K. Chan V. Fearon D. F. Merad M. (2018). Understanding the tumor immune microenvironment (TIME) for effective therapy. Nat. Med. 24 (5), 541–550. 10.1038/s41591-018-0014-x 29686425 PMC5998822

[B12] Brand A. Singer K. Koehl G. E. Kolitzus M. Schoenhammer G. Thiel A. (2016). LDHA-associated lactic acid production blunts tumor immunosurveillance by T and NK cells. Cell Metab. 24 (5), 657–671. 10.1016/j.cmet.2016.08.011 27641098

[B13] Brooks G. A. (2018). The science and translation of lactate shuttle theory. Cell Metab. 27 (4), 757–785. 10.1016/j.cmet.2018.03.008 29617642

[B14] Cascone T. McKenzie J. A. Mbofung R. M. Punt S. Wang Z. Xu C. (2018). Increased tumor glycolysis characterizes immune resistance to adoptive T cell therapy. Cell Metab. 27 (5), 977–987 e974. 10.1016/j.cmet.2018.02.024 29628419 PMC5932208

[B15] Caslin H. L. Abebayehu D. Pinette J. A. Ryan J. J. (2021). Lactate is a metabolic mediator that shapes immune cell fate and function. Front. Physiol. 12, 688485. 10.3389/fphys.2021.688485 34733170 PMC8558259

[B16] Certo M. Llibre A. Lee W. Mauro C. (2022). Understanding lactate sensing and signalling. Trends Endocrinol. Metab. 33 (10), 722–735. 10.1016/j.tem.2022.07.004 35999109

[B17] Chen P. Zuo H. Xiong H. Kolar M. J. Chu Q. Saghatelian A. (2017). Gpr132 sensing of lactate mediates tumor-macrophage interplay to promote breast cancer metastasis. Proc. Natl. Acad. Sci. U. S. A. 114 (3), 580–585. 10.1073/pnas.1614035114 28049847 PMC5255630

[B18] Chen A. N. Luo Y. Yang Y. H. Fu J. T. Geng X. M. Shi J. P. (2021a). Lactylation, a novel metabolic reprogramming code: current status and prospects. Front. Immunol. 12, 688910. 10.3389/fimmu.2021.688910 34177945 PMC8222712

[B19] Chen S. Zhou X. Yang X. Li W. Li S. Hu Z. (2021b). Dual blockade of Lactate/GPR81 and PD-1/PD-L1 pathways enhances the anti-tumor effects of metformin. Biomolecules 11 (9), 1373. 10.3390/biom11091373 34572586 PMC8466555

[B20] Chen L. Huang L. Gu Y. Cang W. Sun P. Xiang Y. (2022). Lactate-lactylation hands between metabolic reprogramming and immunosuppression. Int. J. Mol. Sci. 23 (19), 11943. 10.3390/ijms231911943 36233246 PMC9569569

[B21] Chen J. He G. Cai D. Giovannetti E. Inamura K. Liu S. (2024a). Lactic acid: a narrative review of a promoter of the liver cancer microenvironment. J. Gastrointest. Oncol. 15 (3), 1282–1296. 10.21037/jgo-24-368 38989406 PMC11231854

[B22] Chen S. Xu Y. Zhuo W. Zhang L. (2024b). The emerging role of lactate in tumor microenvironment and its clinical relevance. Cancer Lett. 590, 216837. 10.1016/j.canlet.2024.216837 38548215

[B23] Chen Y. Wu J. Zhai L. Zhang T. Yin H. Gao H. (2024c). Metabolic regulation of homologous recombination repair by MRE11 lactylation. Cell 187 (2), 294–311 e221. 10.1016/j.cell.2023.11.022 38128537 PMC11725302

[B24] Chen J. Huang Z. Chen Y. Tian H. Chai P. Shen Y. (2025). Lactate and lactylation in cancer. Signal Transduct. Target Ther. 10 (1), 38. 10.1038/s41392-024-02082-x 39934144 PMC11814237

[B25] Cheng Y. Guo L. (2025). Lactate metabolism and lactylation in kidney diseases: insights into mechanisms and therapeutic opportunities. Ren. Fail 47 (1), 2469746. 10.1080/0886022X.2025.2469746 40012230 PMC11869332

[B26] Cheng Y. He C. Wang M. Ma X. Mo F. Yang S. (2019). Targeting epigenetic regulators for cancer therapy: mechanisms and advances in clinical trials. Signal Transduct. Target Ther. 4, 62. 10.1038/s41392-019-0095-0 31871779 PMC6915746

[B27] Chou C. K. Lee Y. T. Chen S. M. Hsieh C. W. Huang T. C. Li Y. C. (2015). Elevated urinary D-lactate levels in patients with diabetes and microalbuminuria. J. Pharm. Biomed. Anal. 116, 65–70. 10.1016/j.jpba.2015.06.014 26166004

[B28] Claps G. Faouzi S. Quidville V. Chehade F. Shen S. Vagner S. (2022). The multiple roles of LDH in cancer. Nat. Rev. Clin. Oncol. 19 (12), 749–762. 10.1038/s41571-022-00686-2 36207413

[B29] Collins P. (2022). Point-of-care coagulation testing for postpartum haemorrhage. Best. Pract. Res. Clin. Anaesthesiol. 36 (3-4), 383–398. 10.1016/j.bpa.2022.08.002 36513433

[B30] Comito G. Iscaro A. Bacci M. Morandi A. Ippolito L. Parri M. (2019). Lactate modulates CD4(+) T-cell polarization and induces an immunosuppressive environment, which sustains prostate carcinoma progression via TLR8/miR21 axis. Oncogene 38 (19), 3681–3695. 10.1038/s41388-019-0688-7 30664688

[B31] de la Cruz-Lopez K. G. Castro-Munoz L. J. Reyes-Hernandez D. O. Garcia-Carranca A. Manzo-Merino J. (2019). Lactate in the regulation of tumor microenvironment and therapeutic approaches. Front. Oncol. 9, 1143. 10.3389/fonc.2019.01143 31737570 PMC6839026

[B32] DeBerardinis R. J. Mancuso A. Daikhin E. Nissim I. Yudkoff M. Wehrli S. (2007). Beyond aerobic glycolysis: transformed cells can engage in glutamine metabolism that exceeds the requirement for protein and nucleotide synthesis. Proc. Natl. Acad. Sci. U. S. A. 104 (49), 19345–19350. 10.1073/pnas.0709747104 18032601 PMC2148292

[B33] Draoui N. Schicke O. Seront E. Bouzin C. Sonveaux P. Riant O. (2014). Antitumor activity of 7-aminocarboxycoumarin derivatives, a new class of potent inhibitors of lactate influx but not efflux. Mol. Cancer Ther. 13 (6), 1410–1418. 10.1158/1535-7163.MCT-13-0653 24672058

[B34] Du M. Yu T. Zhan Q. Li H. Zou Y. Geng M. (2022). Development of a novel lactate dehydrogenase A inhibitor with potent antitumor activity and immune activation. Cancer Sci. 113 (9), 2974–2985. 10.1111/cas.15468 35722994 PMC9459323

[B35] Emhoff C. A. Messonnier L. A. Horning M. A. Fattor J. A. Carlson T. J. Brooks G. A. (2013). Gluconeogenesis and hepatic glycogenolysis during exercise at the lactate threshold. J. Appl. Physiol. 114 (3), 297–306. 10.1152/japplphysiol.01202.2012 23239870 PMC8846961

[B36] Fabian E. Kramer L. Siebert F. Hogenauer C. Raggam R. B. Wenzl H. (2017). D-lactic acidosis - case report and review of the literature. Z Gastroenterol. 55 (1), 75–82. 10.1055/s-0042-117647 27723911

[B37] Fan Z. Liu Z. Zhang N. Wei W. Cheng K. Sun H. (2023). Identification of SIRT3 as an eraser of H4K16la. iScience 26 (10), 107757. 10.1016/j.isci.2023.107757 37720100 PMC10504495

[B38] Fang Y. Li Z. Yang L. Li W. Wang Y. Kong Z. (2024). Emerging roles of lactate in acute and chronic inflammation. Cell Commun. Signal 22 (1), 276. 10.1186/s12964-024-01624-8 38755659 PMC11097486

[B39] Fang C. Zhou S. Yu K. Zhang L. Lian W. Tie X. (2026). Lactate metabolism and lactylation in cancer: from pathogenesis to therapeutic advances. Signal Transduct. Target Ther. 11 (1), 190. 10.1038/s41392-026-02672-x 42161914 PMC13190781

[B40] Fendt S. M. (2024). 100 years of the Warburg effect: a cancer metabolism endeavor. Cell 187 (15), 3824–3828. 10.1016/j.cell.2024.06.026 39059359

[B41] Feng J. Yang H. Zhang Y. Wei H. Zhu Z. Zhu B. (2017). Tumor cell-derived lactate induces TAZ-dependent upregulation of PD-L1 through GPR81 in human lung cancer cells. Oncogene 36 (42), 5829–5839. 10.1038/onc.2017.188 28604752

[B42] Fiaschi T. Marini A. Giannoni E. Taddei M. L. Gandellini P. De Donatis A. (2012). Reciprocal metabolic reprogramming through lactate shuttle coordinately influences tumor-stroma interplay. Cancer Res. 72 (19), 5130–5140. 10.1158/0008-5472.CAN-12-1949 22850421

[B43] Frauwirth K. A. Riley J. L. Harris M. H. Parry R. V. Rathmell J. C. Plas D. R. (2002). The CD28 signaling pathway regulates glucose metabolism. Immunity 16 (6), 769–777. 10.1016/s1074-7613(02)00323-0 12121659

[B44] Fu Y. Yu J. Li F. Ge S. (2022). Oncometabolites drive tumorigenesis by enhancing protein acylation: from chromosomal remodelling to nonhistone modification. J. Exp. Clin. Cancer Res. 41 (1), 144. 10.1186/s13046-022-02338-w 35428309 PMC9013066

[B45] Gaffney D. O. Jennings E. Q. Anderson C. C. Marentette J. O. Shi T. Schou Oxvig A. M. (2020). Non-enzymatic lysine lactoylation of glycolytic enzymes. Cell Chem. Biol. 27 (2), 206–213 e206. 10.1016/j.chembiol.2019.11.005 31767537 PMC7395678

[B46] Gao Y. Zhou H. Liu G. Wu J. Yuan Y. Shang A. (2022). Tumor microenvironment: lactic acid promotes tumor development. J. Immunol. Res. 2022, 3119375. 10.1155/2022/3119375 35733921 PMC9207018

[B47] Garcia-Canaveras J. C. Chen L. Rabinowitz J. D. (2019). The tumor metabolic microenvironment: lessons from lactate. Cancer Res. 79 (13), 3155–3162. 10.1158/0008-5472.CAN-18-3726 31171526 PMC6606343

[B48] Garg P. Singhal G. Horne D. Kulkarni P. Salgia R. Singhal S. S. (2025). Molecular PET imaging: unlocking the secrets of cancer metabolism. Biochem. Pharmacol. 242 (Pt 3), 117202. 10.1016/j.bcp.2025.117202 40749955

[B49] Gong T. Zheng C. Ou X. Zheng J. Yu J. Chen S. (2022). Glutamine metabolism in cancers: targeting the oxidative homeostasis. Front. Oncol. 12, 994672. 10.3389/fonc.2022.994672 36324588 PMC9621616

[B50] Gong H. Zhong H. Cheng L. Li L. P. Zhang D. K. (2024). Post-translational protein lactylation modification in health and diseases: a double-edged sword. J. Transl. Med. 22 (1), 41. 10.1186/s12967-023-04842-9 38200523 PMC10777551

[B51] Gottfried E. Kunz-Schughart L. A. Ebner S. Mueller-Klieser W. Hoves S. Andreesen R. (2006). Tumor-derived lactic acid modulates dendritic cell activation and antigen expression. Blood 107 (5), 2013–2021. 10.1182/blood-2005-05-1795 16278308

[B52] Gou Z. Sun Q. Li J. (2025). Exploring lactylation and cancer biology: insights from pathogenesis to clinical applications. Front. Cell Dev. Biol. 13, 1598232. 10.3389/fcell.2025.1598232 40584966 PMC12202587

[B53] Guak H. Al Habyan S. Ma E. H. Aldossary H. Al-Masri M. Won S. Y. (2018). Glycolytic metabolism is essential for CCR7 oligomerization and dendritic cell migration. Nat. Commun. 9 (1), 2463. 10.1038/s41467-018-04804-6 29941886 PMC6018630

[B54] Guan X. Rodriguez-Cruz V. Morris M. E. (2019). Cellular uptake of MCT1 inhibitors AR-C155858 and AZD3965 and their effects on MCT-mediated transport of L-Lactate in murine 4T1 breast tumor cancer cells. AAPS J. 21 (2), 13. 10.1208/s12248-018-0279-5 30617815 PMC6466617

[B55] Halford S. Veal G. J. Wedge S. R. Payne G. S. Bacon C. M. Sloan P. (2023). A phase I dose-escalation study of AZD3965, an oral monocarboxylate transporter 1 inhibitor, in patients with advanced cancer. Clin. Cancer Res. 29 (8), 1429–1439. 10.1158/1078-0432.CCR-22-2263 36652553 PMC7614436

[B56] Han S. Bao X. Zou Y. Wang L. Li Y. Yang L. (2023). d-lactate modulates M2 tumor-associated macrophages and remodels immunosuppressive tumor microenvironment for hepatocellular carcinoma. Sci. Adv. 9 (29), eadg2697. 10.1126/sciadv.adg2697 37467325 PMC10355835

[B57] Hao Z. N. Tan X. P. Zhang Q. Li J. Xia R. Ma Z. (2024). Lactate and lactylation: dual regulators of T-Cell-Mediated tumor immunity and immunotherapy. Biomolecules 14 (12), 1646. 10.3390/biom14121646 39766353 PMC11674224

[B58] Hashimoto T. Ushikubo G. Arao N. Hatabi K. Tsubota K. Hosoi Y. (2025). Oxamate, an LDHA inhibitor, inhibits stemness, including EMT and high DNA repair ability, induces senescence, and exhibits radiosensitizing effects in glioblastoma cells. Int. J. Mol. Sci. 26 (12), 5710. 10.3390/ijms26125710 40565174 PMC12193169

[B59] Hayes C. Donohoe C. L. Davern M. Donlon N. E. (2021). The oncogenic and clinical implications of lactate induced immunosuppression in the tumour microenvironment. Cancer Lett. 500, 75–86. 10.1016/j.canlet.2020.12.021 33347908

[B60] He Y. Song T. Ning J. Wang Z. Yin Z. Jiang P. (2024). Lactylation in cancer: mechanisms in tumour biology and therapeutic potentials. Clin. Transl. Med. 14 (11), e70070. 10.1002/ctm2.70070 39456119 PMC11511673

[B61] He Y. Huang Y. Peng P. Yan Q. Ran L. (2025). Lactate and lactylation in gastrointestinal cancer: current progress and perspectives. Oncol. Rep. 53 (1), 6. (Review). 10.3892/or.2024.8839 39513579 PMC11574708

[B62] Hernandez-Olmos V. Heering J. Marinescu B. Schermeng T. Ivanov V. V. Moroz Y. S. (2024). Development of a potent and selective G2A (GPR132) agonist. J. Med. Chem. 67 (13), 10567–10588. 10.1021/acs.jmedchem.3c02164 38917049 PMC11249017

[B63] Hu X. Huang X. Yang Y. Sun Y. Zhao Y. Zhang Z. (2024a). Dux activates metabolism-lactylation-MET network during early iPSC reprogramming with Brg1 as the histone lactylation reader. Nucleic Acids Res. 52 (10), 5529–5548. 10.1093/nar/gkae183 38512058 PMC11162783

[B64] Hu Y. He Z. Li Z. Wang Y. Wu N. Sun H. (2024b). Lactylation: the novel histone modification influence on gene expression, protein function, and disease. Clin. Epigenetics 16 (1), 72. 10.1186/s13148-024-01682-2 38812044 PMC11138093

[B65] Hua H. Kong Q. Zhang H. Wang J. Luo T. Jiang Y. (2019). Targeting mTOR for cancer therapy. J. Hematol. Oncol. 12 (1), 71. 10.1186/s13045-019-0754-1 31277692 PMC6612215

[B66] Ippolito L. Morandi A. Giannoni E. Chiarugi P. (2019). Lactate: a metabolic driver in the tumour landscape. Trends Biochem. Sci. 44 (2), 153–166. 10.1016/j.tibs.2018.10.011 30473428

[B67] Jiang P. Ning W. Shi Y. Liu C. Mo S. Zhou H. (2021). FSL-Kla: a few-shot learning-based multi-feature hybrid system for lactylation site prediction. Comput. Struct. Biotechnol. J. 19, 4497–4509. 10.1016/j.csbj.2021.08.013 34471495 PMC8385177

[B68] Jiang M. Wang Y. Zhao X. Yu J. (2024). From metabolic byproduct to immune modulator: the role of lactate in tumor immune escape. Front. Immunol. 15, 1492050. 10.3389/fimmu.2024.1492050 39654883 PMC11625744

[B69] Jin J. Bai L. Wang D. Ding W. Cao Z. Yan P. (2023). SIRT3-dependent delactylation of cyclin E2 prevents hepatocellular carcinoma growth. EMBO Rep. 24 (5), e56052. 10.15252/embr.202256052 36896611 PMC10157311

[B70] Jing F. Zhang J. Zhang H. Li T. (2025). Unlocking the multifaceted molecular functions and diverse disease implications of lactylation. Biol. Rev. Camb Philos. Soc. 100 (1), 172–189. 10.1111/brv.13135 39279350

[B71] Lai F. L. Gao F. (2023). Auto-Kla: a novel web server to discriminate lysine lactylation sites using automated machine learning. Brief. Bioinform 24 (2), bbad070. 10.1093/bib/bbad070 36869843

[B72] Lam S. F. Bishop K. W. Mintz R. Fang L. Achilefu S. (2021). Calcium carbonate nanoparticles stimulate cancer cell reprogramming to suppress tumor growth and invasion in an organ-on-a-chip system. Sci. Rep. 11 (1), 9246. 10.1038/s41598-021-88687-6 33927272 PMC8084943

[B73] Li H. Li X. Liu S. Guo L. Zhang B. Zhang J. (2017). Programmed cell death-1 (PD-1) checkpoint blockade in combination with a mammalian target of rapamycin inhibitor restrains hepatocellular carcinoma growth induced by hepatoma cell-intrinsic PD-1. Hepatology 66 (6), 1920–1933. 10.1002/hep.29360 28732118

[B74] Li W. Tanikawa T. Kryczek I. Xia H. Li G. Wu K. (2018). Aerobic glycolysis controls myeloid-derived suppressor cells and tumor immunity via a specific CEBPB isoform in triple-negative breast cancer. Cell Metab. 28 (1), 87–103 e106. 10.1016/j.cmet.2018.04.022 29805099 PMC6238219

[B75] Li X. Yang Y. Zhang B. Lin X. Fu X. An Y. (2022). Lactate metabolism in human health and disease. Signal Transduct. Target Ther. 7 (1), 305. 10.1038/s41392-022-01151-3 36050306 PMC9434547

[B76] Li Y. Wei Y. Huang Y. Qin G. Zhao C. Ren J. (2023). Lactate-responsive gene editing to synergistically enhance macrophage-mediated cancer immunotherapy. Small 19 (35), e2301519. 10.1002/smll.202301519 37156740

[B77] Li Y. Cao Q. Hu Y. He B. Cao T. Tang Y. (2024a). Advances in the interaction of glycolytic reprogramming with lactylation. Biomed. Pharmacother. 177, 116982. 10.1016/j.biopha.2024.116982 38906019

[B78] Li Z. Munim M. B. Sharygin D. A. Bevis B. J. Vander Heiden M. G. (2024b). Understanding the warburg effect in cancer. Cold Spring Harb. Perspect. Med. 15, a041532. 10.1101/cshperspect.a041532 PMC12667404 39284669

[B79] Li J. Ma P. Liu Z. Xie J. (2025). L- and D-Lactate: unveiling their hidden functions in disease and health. Cell Commun. Signal 23 (1), 134. 10.1186/s12964-025-02132-z 40075490 PMC11905701

[B80] Lin X. Zhang F. Bradbury C. M. Kaushal A. Li L. Spitz D. R. (2003). 2-Deoxy-D-glucose-induced cytotoxicity and radiosensitization in tumor cells is mediated via disruptions in thiol metabolism. Cancer Res. 63 (12), 3413–3417. 12810678

[B81] Liu H. Pan M. Liu M. Zeng L. Li Y. Huang Z. (2024a). Lactate: a rising star in tumors and inflammation. Front. Immunol. 15, 1496390. 10.3389/fimmu.2024.1496390 39660139 PMC11628389

[B82] Liu X. Li S. Cui Q. Guo B. Ding W. Liu J. (2024b). Activation of GPR81 by lactate drives tumour-induced cachexia. Nat. Metab. 6 (4), 708–723. 10.1038/s42255-024-01011-0 38499763 PMC11052724

[B83] Liu H. Wang S. Wang J. Guo X. Song Y. Fu K. (2025). Energy metabolism in health and diseases. Signal Transduct. Target Ther. 10 (1), 69. 10.1038/s41392-025-02141-x 39966374 PMC11836267

[B84] Llibre A. Kucuk S. Gope A. Certo M. Mauro C. (2025). Lactate: a key regulator of the immune response. Immunity 58 (3), 535–554. 10.1016/j.immuni.2025.02.008 40073846

[B85] Long Y. Gao Z. Hu X. Xiang F. Wu Z. Zhang J. (2018). Downregulation of MCT4 for lactate exchange promotes the cytotoxicity of NK cells in breast carcinoma. Cancer Med. 7 (9), 4690–4700. 10.1002/cam4.1713 30051648 PMC6143925

[B86] Looyens C. Giraud R. Neto Silva I. Bendjelid K. (2021). Burkitt lymphoma and lactic acidosis: a case report and review of the literature. Physiol. Rep. 9 (4), e14737. 10.14814/phy2.14737 33611854 PMC7897451

[B87] Lu Z. Zheng X. Shi M. Yin Y. Liang Y. Zou Z. (2024). Lactylation: the emerging frontier in post-translational modification. Front. Genet. 15, 1423213. 10.3389/fgene.2024.1423213 38993478 PMC11236606

[B88] Lv H. Dao F. Y. Lin H. (2022). DeepKla: an attention mechanism-based deep neural network for protein lysine lactylation site prediction. Imeta 1 (1), e11. 10.1002/imt2.11 38867734 PMC10989745

[B89] Ma J. Tang L. Xiao J. Tang K. Zhang H. Huang B. (2025). Burning lactic acid: a road to revitalizing antitumor immunity. Front. Med. 19 (3), 456–473. 10.1007/s11684-025-1126-6 40119026

[B90] Ma H. Gu Z. Wang Z. (2026). Lactate and lactylation: metabolic architects of tumor progression and metastasis. Cell Oncol. (Dordr) 49 (2), 66. 10.1007/s13402-026-01182-w 41945221 PMC13057061

[B91] Manoharan I. Prasad P. D. Thangaraju M. Manicassamy S. (2021). Lactate-dependent regulation of immune responses by dendritic cells and macrophages. Front. Immunol. 12, 691134. 10.3389/fimmu.2021.691134 34394085 PMC8358770

[B92] Manosalva C. Quiroga J. Hidalgo A. I. Alarcon P. Anseoleaga N. Hidalgo M. A. (2021). Role of lactate in inflammatory processes: friend or foe. Front. Immunol. 12, 808799. 10.3389/fimmu.2021.808799 35095895 PMC8795514

[B93] Marciniak M. Wagner M. (2023). Innate lymphoid cells and tumor-derived lactic acid: novel contenders in an enduring game. Front. Immunol. 14, 1236301. 10.3389/fimmu.2023.1236301 37868977 PMC10585168

[B94] Mendler A. N. Hu B. Prinz P. U. Kreutz M. Gottfried E. Noessner E. (2012). Tumor lactic acidosis suppresses CTL function by inhibition of p38 and JNK/c-Jun activation. Int. J. Cancer 131 (3), 633–640. 10.1002/ijc.26410 21898391

[B95] Moreno-Yruela C. Zhang D. Wei W. Baek M. Liu W. Gao J. (2022). Class I histone deacetylases (HDAC1-3) are histone lysine delactylases. Sci. Adv. 8 (3), eabi6696. 10.1126/sciadv.abi6696 35044827 PMC8769552

[B96] Mossmann D. Park S. Hall M. N. (2018). mTOR signalling and cellular metabolism are mutual determinants in cancer. Nat. Rev. Cancer 18 (12), 744–757. 10.1038/s41568-018-0074-8 30425336

[B97] Nakajima E. C. Van Houten B. (2013). Metabolic symbiosis in cancer: refocusing the warburg lens. Mol. Carcinog. 52 (5), 329–337. 10.1002/mc.21863 22228080 PMC9972501

[B98] Nguyen N. T. B. Gevers S. Kok R. N. U. Burgering L. M. Neikes H. Akkerman N. (2025). Lactate controls cancer stemness and plasticity through epigenetic regulation. Cell Metab. 37 (4), 903–919 e910. 10.1016/j.cmet.2025.01.002 39933514

[B99] Nunez R. Sidlowski P. F. W. Steen E. A. Wynia-Smith S. L. Sprague D. J. Keyes R. F. (2024). The TRIM33 bromodomain recognizes histone lysine lactylation. ACS Chem. Biol. 19 (12), 2418–2428. 10.1021/acschembio.4c00248 39556662 PMC11706526

[B100] Palucka K. Banchereau J. (2012). Cancer immunotherapy via dendritic cells. Nat. Rev. Cancer 12 (4), 265–277. 10.1038/nrc3258 22437871 PMC3433802

[B101] Pan L. Feng F. Wu J. Fan S. Han J. Wang S. (2022). Demethylzeylasteral targets lactate by inhibiting histone lactylation to suppress the tumorigenicity of liver cancer stem cells. Pharmacol. Res. 181, 106270. 10.1016/j.phrs.2022.106270 35605812

[B102] Peng X. He Y. Huang J. Tao Y. Liu S. (2021). Metabolism of dendritic cells in tumor microenvironment: for immunotherapy. Front. Immunol. 12, 613492. 10.3389/fimmu.2021.613492 33732237 PMC7959811

[B103] Perriello G. Jorde R. Nurjhan N. Stumvoll M. Dailey G. Jenssen T. (1995). Estimation of glucose-alanine-lactate-glutamine cycles in postabsorptive humans: role of skeletal muscle. Am. J. Physiol. 269 (3 Pt 1), E443–E450. 10.1152/ajpendo.1995.269.3.E443 7573421

[B104] Pucino V. Certo M. Bulusu V. Cucchi D. Goldmann K. Pontarini E. (2019). Lactate buildup at the site of chronic inflammation promotes disease by inducing CD4(+) T cell metabolic rewiring. Cell Metab. 30 (6), 1055–1074 e1058. 10.1016/j.cmet.2019.10.004 31708446 PMC6899510

[B105] Qian J. Gong Z. C. Zhang Y. N. Wu H. H. Zhao J. Wang L. T. (2021). Lactic acid promotes metastatic niche formation in bone metastasis of colorectal cancer. Cell Commun. Signal 19 (1), 9. 10.1186/s12964-020-00667-x 33478523 PMC7818572

[B106] Qiao T. Xiong Y. Feng Y. Guo W. Zhou Y. Zhao J. (2021). Inhibition of LDH-A by oxamate enhances the efficacy of Anti-PD-1 treatment in an NSCLC humanized mouse model. Front. Oncol. 11, 632364. 10.3389/fonc.2021.632364 33859941 PMC8042335

[B107] Qu J. Li P. Sun Z. (2023). Histone lactylation regulates cancer progression by reshaping the tumor microenvironment. Front. Immunol. 14, 1284344. 10.3389/fimmu.2023.1284344 37965331 PMC10641494

[B108] Quinn W. J. Jiao J. TeSlaa T. Stadanlick J. Wang Z. Wang L. (2020). Lactate limits T cell proliferation via the NAD(H) redox state. Cell Rep. 33 (11), 108500. 10.1016/j.celrep.2020.108500 33326785 PMC7830708

[B109] Rabinowitz J. D. Enerback S. (2020). Lactate: the ugly duckling of energy metabolism. Nat. Metab. 2 (7), 566–571. 10.1038/s42255-020-0243-4 32694798 PMC7983055

[B110] Rabuka D. Hubbard S. C. Laughlin S. T. Argade S. P. Bertozzi C. R. (2006). A chemical reporter strategy to probe glycoprotein fucosylation. J. Am. Chem. Soc. 128 (37), 12078–12079. 10.1021/ja064619y 16967952 PMC3233198

[B111] Raychaudhuri D. Bhattacharya R. Sinha B. P. Liu C. S. C. Ghosh A. R. Rahaman O. (2019). Lactate induces pro-tumor reprogramming in intratumoral plasmacytoid dendritic cells. Front. Immunol. 10, 1878. 10.3389/fimmu.2019.01878 31440253 PMC6692712

[B112] Renner K. Bruss C. Schnell A. Koehl G. Becker H. M. Fante M. (2019). Restricting glycolysis preserves T cell effector functions and augments checkpoint therapy. Cell Rep. 29 (1), 135–150 e139. 10.1016/j.celrep.2019.08.068 31577944

[B113] Renner O. Mayer M. Leischner C. Burkard M. Berger A. Lauer U. M. (2022). Systematic review of Gossypol/AT-101 in cancer clinical trials. Pharm. (Basel) 15 (2), 144. 10.3390/ph15020144 PMC8879263 35215257

[B114] Robey I. F. Baggett B. K. Kirkpatrick N. D. Roe D. J. Dosescu J. Sloane B. F. (2009). Bicarbonate increases tumor pH and inhibits spontaneous metastases. Cancer Res. 69 (6), 2260–2268. 10.1158/0008-5472.CAN-07-5575 19276390 PMC2834485

[B115] Romero-Garcia S. Moreno-Altamirano M. M. Prado-Garcia H. Sanchez-Garcia F. J. (2016). Lactate contribution to the tumor microenvironment: mechanisms, effects on immune cells and therapeutic relevance. Front. Immunol. 7, 52. 10.3389/fimmu.2016.00052 26909082 PMC4754406

[B116] Ruan D. Hu T. Yang X. Mo X. Ju Q. (2025). Lactate in skin homeostasis: metabolism, skin barrier, and immunomodulation. Front. Immunol. 16, 1510559. 10.3389/fimmu.2025.1510559 40046050 PMC11879785

[B117] Sharma D. Singh M. Rani R. (2022). Role of LDH in tumor glycolysis: regulation of LDHA by small molecules for cancer therapeutics. Semin. Cancer Biol. 87, 184–195. 10.1016/j.semcancer.2022.11.007 36371026

[B118] Sharma K. Kumar M. Dukare A. Vigneshwaran N. Thappa C. Saxena S. (2025). Gossypol and semisynthetic derivatives: chemistry, bioactivities, and mechanism of actions. Chem. Biodivers. 22 (8), e202402872. 10.1002/cbdv.202402872 40145340

[B119] Shestov A. A. Liu X. Ser Z. Cluntun A. A. Hung Y. P. Huang L. (2014). Quantitative determinants of aerobic glycolysis identify flux through the enzyme GAPDH as a limiting step. Elife 3, e03342. 10.7554/eLife.03342 25009227 PMC4118620

[B120] Spinello I. Saulle E. Quaranta M. T. Pasquini L. Pelosi E. Castelli G. (2019). The small-molecule compound AC-73 targeting CD147 inhibits leukemic cell proliferation, induces autophagy and increases the chemotherapeutic sensitivity of acute myeloid leukemia cells. Haematologica 104 (5), 973–985. 10.3324/haematol.2018.199661 30467201 PMC6518905

[B121] Stacpoole P. W. Dirain C. O. (2024). The pyruvate dehydrogenase complex at the epigenetic crossroads of acetylation and lactylation. Mol. Genet. Metab. 143 (1-2), 108540. 10.1016/j.ymgme.2024.108540 39067348

[B122] Su J. Mao X. Wang L. Chen Z. Wang W. Zhao C. (2024). Lactate/GPR81 recruits regulatory T cells by modulating CX3CL1 to promote immune resistance in a highly glycolytic gastric cancer. Oncoimmunology 13 (1), 2320951. 10.1080/2162402X.2024.2320951 38419759 PMC10900271

[B123] Sun X. Wang M. Wang M. Yao L. Li X. Dong H. (2020). Role of proton-coupled monocarboxylate transporters in cancer: from metabolic crosstalk to therapeutic potential. Front. Cell Dev. Biol. 8, 651. 10.3389/fcell.2020.00651 32766253 PMC7379837

[B124] Sun Y. Chen Y. Peng T. (2022). A bioorthogonal chemical reporter for the detection and identification of protein lactylation. Chem. Sci. 13 (20), 6019–6027. 10.1039/d2sc00918h 35685793 PMC9132054

[B125] Sun N. Kabir M. Lee Y. Xie L. Hu X. Velez J. (2023). Discovery of the first lactate dehydrogenase proteolysis targeting chimera degrader for the treatment of pancreatic cancer. J. Med. Chem. 66 (1), 596–610. 10.1021/acs.jmedchem.2c01505 36538511 PMC9969998

[B126] Sun P. Ma L. Lu Z. (2024). Lactylation: linking the warburg effect to DNA damage repair. Cell Metab. 36 (8), 1637–1639. 10.1016/j.cmet.2024.06.015 39111282

[B127] Sun F. Li W. Du R. Liu M. Cheng Y. Ma J. (2025). Impact of glycolysis enzymes and metabolites in regulating DNA damage repair in tumorigenesis and therapy. Cell Commun. Signal 23 (1), 44. 10.1186/s12964-025-02047-9 39849559 PMC11760674

[B128] Tan Y. Xue R. Pan Y. He Z. Hu X. Li Y. (2026). Cancer cachexia: molecular basis and therapeutic advances. Signal Transduct. Target Ther. 11 (1), 16. 10.1038/s41392-025-02331-7 41526343 PMC12795897

[B129] Tao H. Zhong X. Zeng A. Song L. (2023). Unveiling the veil of lactate in tumor-associated macrophages: a successful strategy for immunometabolic therapy. Front. Immunol. 14, 1208870. 10.3389/fimmu.2023.1208870 37564659 PMC10411982

[B130] van Hall G. (2010). Lactate kinetics in human tissues at rest and during exercise. Acta Physiol. (Oxf) 199 (4), 499–508. 10.1111/j.1748-1716.2010.02122.x 20345411

[B131] Varner E. L. Trefely S. Bartee D. von Krusenstiern E. Izzo L. Bekeova C. (2020). Quantification of lactoyl-CoA (lactyl-CoA) by liquid chromatography mass spectrometry in mammalian cells and tissues. Open Biol. 10 (9), 200187. 10.1098/rsob.200187 32961073 PMC7536085

[B132] Vaupel P. Schmidberger H. Mayer A. (2019). The Warburg effect: essential part of metabolic reprogramming and central contributor to cancer progression. Int. J. Radiat. Biol. 95 (7), 912–919. 10.1080/09553002.2019.1589653 30822194

[B133] Vavricka J. Broz P. Follprecht D. Novak J. Krouzecky A. (2024). Modern perspective of lactate metabolism. Physiol. Res. 73 (4), 499–514. 10.33549/physiolres.935331 39264074 PMC11414593

[B134] Wada H. Hamaguchi R. Narui R. Morikawa H. (2022). Meaning and significance of Alkalization therapy for cancer. Front. Oncol. 12, 920843. 10.3389/fonc.2022.920843 35965526 PMC9364696

[B135] Walid M. Saboor M. Haque S. Mohammad M. G. (2026). The tumor microenvironment in hematologic malignancies: immune evasion, metabolic reprogramming, and therapeutic resistance. Blood Sci. 8 (1), e00270. 10.1097/BS9.0000000000000270 41726040 PMC12919749

[B136] Wan N. Wang N. Yu S. Zhang H. Tang S. Wang D. (2022). Cyclic immonium ion of lactyllysine reveals widespread lactylation in the human proteome. Nat. Methods 19 (7), 854–864. 10.1038/s41592-022-01523-1 35761067

[B137] Wan L. Zhang H. Liu J. He Q. Zhao J. Pan C. (2025). Lactylation and human disease. Expert Rev. Mol. Med. 27, e10. 10.1017/erm.2025.3 39895568 PMC11879378

[B138] Wang J. X. Choi S. Y. C. Niu X. Kang N. Xue H. Killam J. (2020). Lactic acid and an acidic tumor microenvironment suppress anticancer immunity. Int. J. Mol. Sci. 21 (21), 8363. 10.3390/ijms21218363 33171818 PMC7664620

[B139] Wang Y. Qin L. Chen W. Chen Q. Sun J. Wang G. (2021a). Novel strategies to improve tumour therapy by targeting the proteins MCT1, MCT4 and LAT1. Eur. J. Med. Chem. 226, 113806. 10.1016/j.ejmech.2021.113806 34517305

[B140] Wang Z. H. Peng W. B. Zhang P. Yang X. P. Zhou Q. (2021b). Lactate in the tumour microenvironment: from immune modulation to therapy. EBioMedicine 73, 103627. 10.1016/j.ebiom.2021.103627 34656878 PMC8524104

[B141] Wang T. Ye Z. Li Z. Jing D. S. Fan G. X. Liu M. Q. (2023). Lactate-induced protein lactylation: a bridge between epigenetics and metabolic reprogramming in cancer. Cell Prolif. 56 (10), e13478. 10.1111/cpr.13478 37060186 PMC10542650

[B142] Wang A. Zou Y. Liu S. Zhang X. Li T. Zhang L. (2024a). Comprehensive multiscale analysis of lactate metabolic dynamics *in vitro* and *in vivo* using highly responsive biosensors. Nat. Protoc. 19 (5), 1311–1347. 10.1038/s41596-023-00948-y 38307980 PMC12812421

[B143] Wang G. Zou X. Chen Q. Nong W. Miao W. Luo H. (2024b). The relationship and clinical significance of lactylation modification in digestive system tumors. Cancer Cell Int. 24 (1), 246. 10.1186/s12935-024-03429-8 39010066 PMC11251390

[B144] Wang J. Wang Z. Wang Q. Li X. Guo Y. (2024c). Ubiquitous protein lactylation in health and diseases. Cell Mol. Biol. Lett. 29 (1), 23. 10.1186/s11658-024-00541-5 38317138 PMC10845568

[B145] Wang W. Wang H. Wang Q. Yu X. Ouyang L. (2024d). Lactate-induced protein lactylation in cancer: functions, biomarkers and immunotherapy strategies. Front. Immunol. 15, 1513047. 10.3389/fimmu.2024.1513047 39867891 PMC11757118

[B146] Wanka C. Brucker D. P. Bahr O. Ronellenfitsch M. Weller M. Steinbach J. P. (2012). Synthesis of cytochrome C oxidase 2: a p53-dependent metabolic regulator that promotes respiratory function and protects glioma and colon cancer cells from hypoxia-induced cell death. Oncogene 31 (33), 3764–3776. 10.1038/onc.2011.530 22120717

[B147] Wculek S. K. Cueto F. J. Mujal A. M. Melero I. Krummel M. F. Sancho D. (2020). Dendritic cells in cancer immunology and immunotherapy. Nat. Rev. Immunol. 20 (1), 7–24. 10.1038/s41577-019-0210-z 31467405

[B148] Weiss H. J. Angiari S. (2020). Metabolite transporters as regulators of immunity. Metabolites 10 (10), 418. 10.3390/metabo10100418 33086598 PMC7603148

[B149] Wu H. Wang Y. Ying M. Jin C. Li J. Hu X. (2021). Lactate dehydrogenases amplify reactive oxygen species in cancer cells in response to oxidative stimuli. Signal Transduct. Target Ther. 6 (1), 242. 10.1038/s41392-021-00595-3 34176927 PMC8236487

[B150] Wu W. Yao H. Lin H. Shi J. (2022). Microbiotic nanomedicine for tumor-specific chemotherapy-synergized innate/adaptive antitumor immunity. Nano Today 42, 101377. 10.1016/j.nantod.2022.101377

[B151] Wu D. Zhang K. Khan F. A. Wu Q. Pandupuspitasari N. S. Tang Y. (2023a). The emerging era of lactate: a rising star in cellular signaling and its regulatory mechanisms. J. Cell Biochem. 124 (8), 1067–1081. 10.1002/jcb.30458 37566665

[B152] Wu H. Huang H. Zhao Y. (2023b). Interplay between metabolic reprogramming and post-translational modifications: from glycolysis to lactylation. Front. Immunol. 14, 1211221. 10.3389/fimmu.2023.1211221 37457701 PMC10338923

[B153] Xie H. Yin J. Shah M. H. Menefee M. E. Bible K. C. Reidy-Lagunes D. (2019). A phase II study of the orally administered negative enantiomer of gossypol (AT-101), a BH3 mimetic, in patients with advanced adrenal cortical carcinoma. Invest New Drugs 37 (4), 755–762. 10.1007/s10637-019-00797-1 31172443 PMC7515770

[B154] Xie B. Lin J. Chen X. Zhou X. Zhang Y. Fan M. (2023). CircXRN2 suppresses tumor progression driven by histone lactylation through activating the Hippo pathway in human bladder cancer. Mol. Cancer 22 (1), 151. 10.1186/s12943-023-01856-1 37684641 PMC10486081

[B155] Xie B. Zhang M. Li J. Cui J. Zhang P. Liu F. (2024). KAT8-catalyzed lactylation promotes eEF1A2-mediated protein synthesis and colorectal carcinogenesis. Proc. Natl. Acad. Sci. U. S. A. 121 (8), e2314128121. 10.1073/pnas.2314128121 38359291 PMC10895275

[B156] Xing B. C. Wang C. Ji F. J. Zhang X. B. (2018). Synergistically suppressive effects on colorectal cancer cells by combination of mTOR inhibitor and glycolysis inhibitor, Oxamate. Int. J. Clin. Exp. Pathol. 11 (9), 4439–4445. 31949841 PMC6962984

[B157] Xu R. H. Pelicano H. Zhang H. Giles F. J. Keating M. J. Huang P. (2005). Synergistic effect of targeting mTOR by rapamycin and depleting ATP by inhibition of glycolysis in lymphoma and leukemia cells. Leukemia 19 (12), 2153–2158. 10.1038/sj.leu.2403968 16193082

[B158] Xu H. Li L. Wang S. Wang Z. Qu L. Wang C. (2023). Royal jelly acid suppresses hepatocellular carcinoma tumorigenicity by inhibiting H3 histone lactylation at H3K9la and H3K14la sites. Phytomedicine 118, 154940. 10.1016/j.phymed.2023.154940 37453194

[B159] Xu B. Liu Y. Li N. Geng Q. (2024). Lactate and lactylation in macrophage metabolic reprogramming: current progress and outstanding issues. Front. Immunol. 15, 1395786. 10.3389/fimmu.2024.1395786 38835758 PMC11148263

[B160] Yang X. Lu Y. Hang J. Zhang J. Zhang T. Huo Y. (2020). Lactate-modulated immunosuppression of myeloid-derived suppressor cells contributes to the radioresistance of pancreatic cancer. Cancer Immunol. Res. 8 (11), 1440–1451. 10.1158/2326-6066.CIR-20-0111 32917658

[B161] Yang K. Fan M. Wang X. Xu J. Wang Y. Tu F. (2022). Lactate promotes macrophage HMGB1 lactylation, acetylation, and exosomal release in polymicrobial sepsis. Cell Death Differ. 29 (1), 133–146. 10.1038/s41418-021-00841-9 34363018 PMC8738735

[B162] Yang Z. Yan C. Ma J. Peng P. Ren X. Cai S. (2023). Lactylome analysis suggests lactylation-dependent mechanisms of metabolic adaptation in hepatocellular carcinoma. Nat. Metab. 5 (1), 61–79. 10.1038/s42255-022-00710-w 36593272

[B163] Yu H. Zhu T. Ma D. Cheng X. Wang S. Yao Y. (2024a). The role of nonhistone lactylation in disease. Heliyon 10 (18), e36296. 10.1016/j.heliyon.2024.e36296 39315193 PMC11417196

[B164] Yu X. Yang J. Xu J. Pan H. Wang W. Yu X. (2024b). Histone lactylation: from tumor lactate metabolism to epigenetic regulation. Int. J. Biol. Sci. 20 (5), 1833–1854. 10.7150/ijbs.91492 38481814 PMC10929197

[B165] Zeng Z. Mukherjee A. Varghese A. P. Yang X. L. Chen S. Zhang H. (2020). Roles of G protein-coupled receptors in inflammatory bowel disease. World J. Gastroenterol. 26 (12), 1242–1261. 10.3748/wjg.v26.i12.1242 32256014 PMC7109274

[B166] Zhang D. Tang Z. Huang H. Zhou G. Cui C. Weng Y. (2019). Metabolic regulation of gene expression by histone lactylation. Nature 574 (7779), 575–580. 10.1038/s41586-019-1678-1 31645732 PMC6818755

[B167] Zhang Y. Zhang X. Meng Y. Xu X. Zuo D. (2022). The role of glycolysis and lactate in the induction of tumor-associated macrophages immunosuppressive phenotype. Int. Immunopharmacol. 110, 108994. 10.1016/j.intimp.2022.108994 35777265

[B168] Zhang T. Chen L. Kueth G. Shao E. Wang X. Ha T. (2024a). Lactate's impact on immune cells in sepsis: unraveling the complex interplay. Front. Immunol. 15, 1483400. 10.3389/fimmu.2024.1483400 39372401 PMC11449721

[B169] Zhang X. Liang C. Wu C. Wan S. Xu L. Wang S. (2024b). A rising star involved in tumour immunity: lactylation. J. Cell Mol. Med. 28 (20), e70146. 10.1111/jcmm.70146 39417674 PMC11483924

[B170] Zhang Y. Song H. Li M. Lu P. (2024c). Histone lactylation bridges metabolic reprogramming and epigenetic rewiring in driving carcinogenesis: oncometabolite fuels oncogenic transcription. Clin. Transl. Med. 14 (3), e1614. 10.1002/ctm2.1614 38456209 PMC10921234

[B171] Zhao L. Qi H. Lv H. Liu W. Zhang R. Yang A. (2025). Lactylation in health and disease: physiological or pathological? Theranostics 15 (5), 1787–1821. 10.7150/thno.105353 39897556 PMC11780532

[B172] Zheng C. Tan H. Niu G. Huang X. Lu J. Chen S. (2025). ACAT1-Mediated ME2 acetylation drives chemoresistance in ovarian cancer by linking glutaminolysis to lactate production. Adv. Sci. (Weinh) 12 (14), e2416467. 10.1002/advs.202416467 39951294 PMC11984883

[B173] Zhou H. C. Xin-Yan Y. Yu W. W. Liang X. Q. Du X. Y. Liu Z. C. (2022). Lactic acid in macrophage polarization: the significant role in inflammation and cancer. Int. Rev. Immunol. 41 (1), 4–18. 10.1080/08830185.2021.1955876 34304685

[B174] Zhou C. Li W. Liang Z. Wu X. Cheng S. Peng J. (2024). Mutant KRAS-activated circATXN7 fosters tumor immunoescape by sensitizing tumor-specific T cells to activation-induced cell death. Nat. Commun. 15 (1), 499. 10.1038/s41467-024-44779-1 38216551 PMC10786880

[B175] Zhu Z. Zheng X. Zhao P. Chen C. Xu G. Ke X. (2025). Potential of lactylation as a therapeutic target in cancer treatment. Mol. Med. Rep. 31 (4), 91. (Review). 10.3892/mmr.2025.13456 39950331 PMC11836599

[B176] Zong Z. Xie F. Wang S. Wu X. Zhang Z. Yang B. (2024). Alanyl-tRNA synthetase, AARS1, is a lactate sensor and lactyltransferase that lactylates p53 and contributes to tumorigenesis. Cell 187 (10), 2375–2392 e2333. 10.1016/j.cell.2024.04.002 38653238

